# Non-specific increase in alpha power during a neurofeedback session targeting its downregulation

**DOI:** 10.1162/IMAG.a.1258

**Published:** 2026-05-29

**Authors:** Jacob Maaz, Alexandra Dia, Laurent Waroquier, Véronique Paban, Arnaud Rey

**Affiliations:** Aix Marseille Univ, CNRS, CRPN, Marseille, France; Institute Neuro-Marseille, Aix Marseille Univ, Marseille, France; Institute of Language, Communication and the Brain, Aix Marseille Univ, Marseille, France; Aix Marseille Univ, PSYCLE, Aix-en-Provence, France

**Keywords:** EEG, neurofeedback, non-specific influences, alpha activity

## Abstract

Electroencephalographic neurofeedback is often assumed to provide volitional control over neural oscillations, with the alpha rhythm regarded as a particularly accessible target. Yet, evidence supporting single-session alpha modulation remains controversial, largely because of insufficient controls for non-specific influences such as time-on-task effects. This study examined whether individuals can downregulate alpha parietal-scalp power during a single neurofeedback session while controlling for feedback veracity and targeted modulation direction. Healthy individuals completed three training blocks in which the size of a visual stimulus reflected either their real-time alpha power (Alpha-Down group), alpha power in the opposite direction (Alpha-Up group), or prerecorded trajectories (Sham group), followed by a transfer block without feedback. The frequency of feedback update was varied across training blocks (1, 5, or 10 *Hz*). Results showed robust within-session increases in alpha power across all groups, independent of targeted direction, feedback veracity, or update frequency. Theta and sensorimotor rhythm bands also demonstrated an independent increase, while beta remained stable. These findings indicate that apparent alpha modulation in single-session protocols reflects spontaneous, non-specific increases in oscillatory activity rather than genuine volitional control. The results generalise previous evidence on alpha upregulation to its attempted downregulation, reinforcing that time-dependent dynamics such as arousal, fatigue, or mind wandering may dominate single-session outcomes. This work highlights the need for stringent methodological controls when evaluating the specificity of neurofeedback interventions.

## 1 Introduction

Electroencephalographic neurofeedback (EEG-NF) is a closed-loop training approach in which individuals learn to volitionally modulate neural oscillations (Sitaram et al., 2017). By repeatedly coupling real-time brain activity to an explicit sensory feedback, EEG-NF aims to shape self-regulation skills that can generalise beyond the training context towards clinical or behavioural improvements (Ros et al., 2020; Strehl, 2014). Among EEG rhythms, alpha oscillations (8–12 *Hz*) have been a central target because of their robust prevalence in the EEG signal and their established link to perceptual, attentional, and memory processes (Jensen, 2024; Klimesch, 2012; Rogala et al., 2016). As a result, alpha activity is considered to be easily modulated by EEG-NF, even during single-session protocols (Agnoli et al., 2018; Belinskaia et al., 2020; Chikhi et al., 2023; Escolano et al., 2014; Ros et al., 2010). Alpha modulation has been applied to both improve healthy cognitive functioning (Berger & Davelaar, 2018; Hanslmayr et al., 2005; Naas et al., 2019) and treat several clinical conditions such as anxio-depressive and post-traumatic stress disorders (Hardt & Kamiya, 1978; Kluetsch et al., 2014; Peeters et al., 2014).

Despite this conceptual appeal, EEG-NF currently faces criticism regarding the specificity of its modulatory effects on brain, clinical and behavioural outcomes (McGough, 2022; Thibault & Raz, 2017). One core EEG-NF assumption is that providing real-time feedback enables participants to achieve active EEG self-regulation which, in turn, is specifically responsible for targeted clinical or behavioural improvements. However, robust studies have instead established that multiple non-specific confounds (e.g., general neuroscientific context, expectancies, motivation, and/or placebo-like effects) drive these improvements during EEG-NF interventions (Dessy et al., 2018; Neurofeedback Collaborative Group, 2021; Schabus et al., 2017; Schönenberg et al., 2017, 2021). Furthermore, these diverse non-specific confounds have been argued to influence brain outcomes as well (Maaz et al., 2025; Witte et al., 2018). In particular, the alpha band power is known to exhibit positive non-stationarities at multiple timescales (Benwell et al., 2019; Cohen, 2014; Maaz, Paban, et al., 2026). Notably, these increases in alpha power may be attributed to non-specific phenomena that are influenced by time-on-task in traditional EEG experiments. These include fluctuations in arousal, fatigue and mind wandering occurrence (Arnau et al., 2020; Compton et al., 2019). However, the majority of alpha EEG-NF studies lack proper controls to address non-specific influences on outcomes (Chiasson et al., 2023; Rogala et al., 2016). As a result, previous alpha upregulation during single EEG-NF sessions might be inadequately attributed to the historical hypothesis of EEG volitional control (e.g., Micoulaud Franchi et al., 2020).

Accordingly, our group recently conducted a randomised sham-controlled study to evaluate the specificity of single-session alpha upregulation (Maaz, Waroquier, et al., 2026). Using a double-blind design, participants were randomly divided into two groups. Both groups completed the same EEG-NF training with identical instructions. During training, the diameter of a circle was updated in real time to provide visual feedback. This update depended either on participant’s ongoing alpha power (i.e., Alpha-Up group which aimed alpha upregulation) or on prerecorded alpha trajectories (i.e., so-called Sham procedure, see Sorger et al., 2019). Importantly, alpha power increased throughout the session irrespective of feedback veracity. Furthermore, subsequent comparisons between the Sham group and a complementary passive group (i.e., instructed only to visualise random fluctuations of the same circle size) revealed parallel similar alpha time courses. These results indicate that, under standard single-session settings, apparent alpha upregulation can be explained by general, non-specific drifts that are unrelated to the EEG-NF procedure or the engagement in self-regulation. They call into question prior claims of specific volitional alpha enhancement that did not fully neutralise non-specific confounds such as general time-on-task effects.

Similarly to alpha upregulation, a separate line of work reported that alpha power can be effectively reduced within single EEG-NF sessions (Kluetsch et al., 2014; Nan et al., 2020; Nowlis & Kamiya, 1970; Ros et al., 2010, 2013). Yet, these previous studies were limited by important methodological parameters such as the absence of control group or condition. A sham-controlled group was solely implemented by Ros et al. (2013) and Nan et al. (2020), who effectively concluded on a superior alpha downregulation in the experimental group (*vs*. sham). Nevertheless, sham-controlled studies are known to be very time consuming and resource consuming (e.g., Chikhi et al., 2024). As a result, these studies suffered from very low sample sizes, a limitation that considerably enhances Type I error rates (Button et al., 2013). Thus, whether individuals can volitionally downregulate alpha power still remains to be determined in better powered studies.

**Fig. 1.**
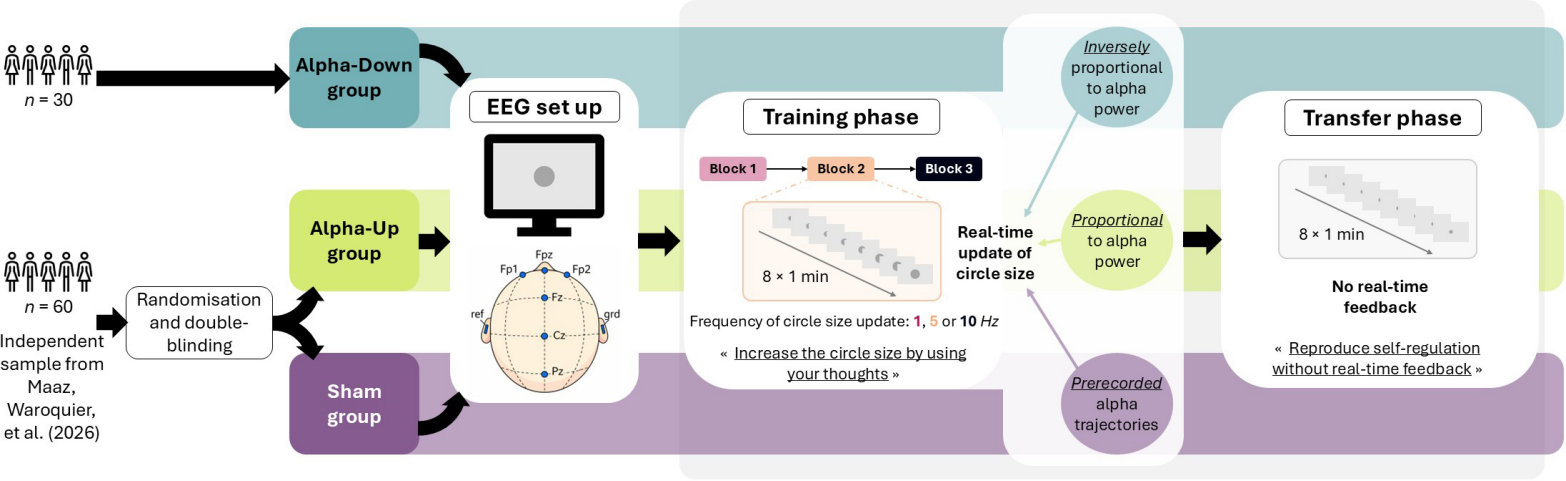
Overview of current experimental design. The present study involved an **Alpha-Down** group of heathy young adults (*n* = 30) undergoing a single EEG neurofeedback (EEG-NF) session which aimed the downregulation of alpha (8–12 *Hz*) power at Pz. After initial EEG installation, the EEG-NF session comprised a **Training phase** and a **Transfer phase**. The **Training phase** was composed of three blocks, and each block consisted of eight 1-minute trials. During each trial, participants were presented with a grey circle at the centre of a screen. The circle size was continuously updated as real-time feedback of participants’ alpha power. Participants were instructed to find mental strategies that effectively increase, as much as possible, the circle size by using their thoughts. As the circle size was **inversely proportional** to their real-time alpha power, the aim was to learn to decrease as much as possible their alpha power by increasing the circle size during training. Across the three training blocks, the frequency of circle size update was within subject manipulated at either **1, 5, or 10 *****Hz*** (i.e., update every 1000, 200, or 100 milliseconds, respectively). The transfer phase was composed of one block of eight 1-minute trials. During this block, the circle size remained fixed throughout. Participants were instructed to reproduce the mental strategies that effectively increased the circle size during training. To rigorously evaluate the effectiveness of the present study to downregulate alpha power, alpha trajectories of the **Alpha-Down** group were systematically compared with an **Alpha-Up** (*n* = 30) and a **Sham** (*n* = 30) groups drawn from an independent study (Maaz, Waroquier, et al., 2026). This study implemented a double-blind sham-controlled procedure aiming to dissociate specific volitional EEG self-regulation from non-specific influences on the single-session upregulation of alpha power. Participants from these **Alpha-Up** and **Sham** groups underwent through an identical procedure, with the same instructions and materials as those from the current **Alpha-Down** group. The only between-group difference was the nature of the real-time circle size update during training. Compared with the **Alpha-Down** group in which the circle size was *inversely* proportional to participants’ real-time alpha power, the circle size was *positively* proportional to participants’ real-time alpha power in the **Alpha-Up** group. In the **Sham** group, the circle size update did not rely on participants’ real-time EEG activity, but rather on alpha power trajectories prerecorded during another independent study (Maaz, Paban, et al., 2026).

Addressing these methodological limitations, the present study rigorously tests whether, under conditions with substantial statistical power, individuals can volitionally downregulate their alpha power within a single EEG-NF session. The alpha trajectories of one Alpha-Down group were compared with those of independent Alpha-Up and Sham groups from our previous study (Maaz, Waroquier, et al., 2026), enabling a stringent evaluation of specificity against both genuine but opposite-direction feedback and non-contingent, that is, sham feedback (Sorger et al., 2019), respectively. Additionally, to match prior study design, the frequency of feedback update (either at 1, 5, or 10 *Hz*) was manipulated within participants across training blocks, thereby evaluating whether feedback update timing affects alpha modulation performance. Following training, the transfer of self-regulation performance was also assessed during without-feedback trials (Enriquez-Geppert et al., 2017; Strehl, 2014). Figure 1 presents a schematic illustration of the current experimental design.

We hypothesised that alpha power should steadily increase during both training and transfer phases. Furthermore, this increase should be independent of the frequency of feedback update (Maaz, Paban, et al., 2026; Maaz, Waroquier, et al., 2026; Maaz et al., 2025), the targeted direction of alpha regulation (i.e., Alpha-Up *vs*. Alpha-Down), and the veracity of the feedback (i.e., Alpha-Down *vs.* Sham; Benwell et al., 2019; Maaz, Waroquier, et al., 2026). To address neurophysiological specificity issues (Ros et al., 2020), we also evaluated each effect on the spectral power of other frequency bands frequently targeted by EEG-NF, that is, theta (4–8 *Hz*), sensorimotor rhythm (SMR, 12–15 *Hz*), and beta (15–30 *Hz*).

## 2 Method

We combined and analysed EEG data from three groups of participants who underwent very similar experimental conditions. This method specifically applies to the Alpha-Down group of the present study. The remaining Alpha-Up and Sham groups were part of a previous double-blind sham-controlled study of our group. Further details about this study can be found in the original paper (Maaz, Waroquier, et al., 2026).

### 2.1 Participants

The sample size of the present study was determined using a Bayesian a priori power analysis for three group comparisons in such an experimental design (for a similar approach, see Nalborczyk et al., 2023). Simulated datasets were generated to mirror the current experimental structure, matching the planned statistical analyses (cf. Equation 1). The number of participants per group was manipulated between simulated datasets (24, 30, 36, 42, or 48). For each sample size, 1000 simulations were performed under a conservative scenario that all model parameters were null. This was done because confirming the absence of effects (i.e., the null hypothesis) necessitates larger samples than detecting small, medium, or large effects (Schönbrodt & Wagenmakers, 2018; Schönbrodt et al., 2017). Therefore, statistical power was defined as the averaged probability of obtaining a Bayes Factor of 10 or greater favouring the null hypothesis. A sample size of 24 per group was judged sufficient to obtain enough statistical power (i.e., averaged probability superior to 0.80). As a result, 30 healthy young adults were included in the current Alpha-Down group (*M_age_* = 22.22 years, *SD* = 5.01, age range = 17–38; 21 females; 27 right-handed). Adding the Alpha-Up (*n* = 30; *M_age_* = 22.77 years, *SD* = 3.42, age range = 19–30; 22 females; 24 right-handed) and Sham (*n* = 30; *M_age_* = 22.63 years, *SD* = 3.06, age range = 19–30; 28 females; 27 right-handed) groups (Maaz, Waroquier, et al., 2026), this resulted in 90 participants in total.

All participants reported no history of neurological or psychiatric disorders, as well as normal or corrected-to-normal vision. Recruitment was carried out via a university learning platform or internal laboratory channels. Student participants (*n* = 41) received course credits as compensation. Written informed consent was obtained from all participants in accordance with the Declaration of Helsinki. To ensure anonymity, a unique identification code was assigned to each participant. The study received ethical approval from the French Personal Protection Committee (CPP Sud Méditerranée V, ref. 19.09.12.44636).

### 2.2 Material and neurofeedback implementation

Participants completed an EEG-NF session composed of four blocks. Each block comprised eight 60-second trials. During each trial, a grey circle was presented at the centre of a screen. First, during three training blocks, the circle size was continuously updated as real-time feedback of the participant’s alpha band (8–12 *Hz*) power at Pz (Chikhi et al., 2023). The circle size was inversely proportional to real-time alpha power. To target alpha power downregulation, participants were instructed to maximise the circle size as much as possible, therefore, mirroring the previous procedure of Alpha-Up and Sham groups (Maaz, Waroquier, et al., 2026). Between the three training blocks, the frequency of feedback update was manipulated at 1, 5, and 10 *Hz*, reflecting common update frequencies in EEG-NF protocols (Berger & Davelaar, 2018; Boe et al., 2014; Enriquez-Geppert, Huster, Figge, et al., 2014; Enriquez-Geppert, Huster, Scharfenort, et al., 2014; Hsueh et al., 2016; Kober et al., 2015; Salari et al., 2014; Studer et al., 2014; Wei et al., 2017). To counterbalance the block order across subjects, a Latin-square design was employed. All six possible triplets were generated while ensuring each frequency appears in every temporal position (123, 132, 213, 231, 312, 321) and an even distribution across participants. After the training phase, a fourth transfer block evaluated alpha downregulation transfer. In this block, the circle size remained constant (100 pixels) across trials. The aim was to assess participants’ ability to self-regulate their EEG activity without the help of the real-time feedback (e.g., Strehl, 2014). Between the trials of all blocks, participants were free to take self-paced breaks. The break length was not measured but typically lasted few seconds to a couple of minutes depending on the participant.

At the end of the session, participants responded to two questions: (i) “During the first three training blocks, to what extent did you feel in control of the variations of the circle?”; and (ii) “It is possible that the feedback which has been presented to you during the training was actually random. To what extent do you believe that the feedback was random?”. Participants answered through a 5-point Likert scale (1—“Not at all” to 5—“Completely”). These questions aimed to assess the participants’ feeling of feedback control and belief of having received sham feedback, respectively. Importantly, these subjective measures are recommended for implementation in sham-controlled EEG-NF studies to control for differences in feedback and blinding credibility between groups (Ros et al., 2020).

### 2.3 EEG recording

EEG was digitalised at a sample rate of 250 *Hz*, in microvolts (*µV*), in Matlab Release 2023a (Mathworks, Inc.) and using Brainflow library version 5-8-1 with the OpenBCI Cyton 8-channels board. Data acquisition was handled via the laptop’s GPU to reduce computation time. EEG signals were recorded from six OpenBCI Gold Cup electrodes positioned according to the 10–20 International System: Fp1, Fpz, Fp2, Fz, Cz, and Pz. Two OpenBCI Earclip electrodes were placed on the left and the right earlobes, serving, respectively, as a reference and as a noise-cancelling ground. Electrode impedance was maintained below 10 *kΩ*.

### 2.4 Procedure

Participants were seated in front of a flat-screen monitor (screen resolution: 1920 × 1080 pixels; screen size: 52.704 × 29.646 *cm*; refresh rate: 60 *Hz*). The distance between the monitor and the back of the chair was kept constant (90–100 *cm*). EEG setup was installed and impedance checked after obtaining written and informed consent. The EEG-NF session was implemented using Psychtoolbox-3 (Kleiner et al., 2007) and comprised a training (three blocks) and a transfer (one block) phases. Participants were first verbally informed about the structure of the training phase and instructed to increase as much as possible the circle size by using their thoughts. Participants were not informed about the specific EEG-NF target (i.e., downregulate their alpha power at Pz), nor provided with a pre-defined mental strategy. Rather, they were encouraged to find mental strategies correlating with increases in circle size as in common practices (Enriquez-Geppert et al., 2017). Prior to the transfer block, participants were also provided with verbal instructions encouraging them to reproduce, without continuous feedback, the mental strategy used to increase the circle size during training. Supplementary Table S1 presents the exact verbal instructions provided to participants before the training and the transfer phases. Finally, after the transfer block, questions were presented to the participant for qualitative purposes (cf. Material and neurofeedback implementation section).

Throughout the session, participants were instructed to remain as calm and relaxed as possible to avoid introducing artefacts into the EEG signal. They had to initiate each trial by pressing the “Enter” key on a keyboard. Experimenters remained in the same room during the session but stayed out of the participant’s sight. The EEG-NF session was conducted in a quiet laboratory room with constant artificial lighting (identical for all groups). They were scheduled either in the morning or in the afternoon depending on participant availability. Ten out of 30 participants in the Alpha-Down group underwent testing in the morning. This ratio was similar in the Alpha-Up (13/30) and Sham (11/30) groups.

### 2.5 EEG online processing

During the trials of the training phase, a real-time time–frequency analysis (moving-window short-FFT) was performed in Matlab R2023a and followed Keil et al. (2022)’s recommendations (see Supplementary Table S2 for the corresponding completed checklist) to obtain the spectral power of the alpha frequency band (8–12 *Hz*) at Pz. At each step of the analysis, zero-phase filtering was first applied using a 1–20 Hz bandpass filter (4^th^ order IIR Butterworth) with the Matlab *filtfilt* function. Then, to compute spectral power estimates in decibels (*dB*), a symmetric Hann window of 500 samples (2-second length) was used with the Matlab *pspectrum* function. Alpha spectral power was obtained by averaging these estimates within the 8–12 *Hz* range.

Between the three training blocks, different frequencies for feedback update were used: 1, 5, or 10 *Hz*. To match the corresponding frequency, the overlap between two consecutive windows was determined by subtracting the EEG sampling rate (250 *Hz*) divided by the feedback update frequency from the window length (500 samples). This, respectively, resulted in window overlaps of 250 (500–250/1), 450 (500–250/5), and 475 (500–250/10) samples (i.e., 50%, 90%, and 95%, respectively). Across blocks and participants, the averaged technical delay due to EEG online processing (i.e., after EEG acquisition and before feedback update) was 29 *ms*.

### 2.6 EEG offline processing

Offline processing of EEG data was performed in Matlab R2023a according to Keil et al. (2022) guidelines (see Supplementary Table S3 for the completed checklist). The spectral power of theta (4–8 *Hz*), alpha (8–12 *Hz*), SMR (12–15 *Hz*), and beta (15–30 *Hz*) frequency bands was computed through in-house Matlab scripts and EEGLAB (Delorme & Makeig, 2004). Neurofeedback studies typically lack an explicit model for the generation of oscillatory activity and the 1/*f* noise (Enriquez-Geppert et al., 2017). We, therefore, adopted the narrowband model implicitly assumed in the field.

First, all channel data were zero-phase filtered (Matlab *filtfilt* function) using a 0.5 *Hz* high-pass filter (6^th^ order IIR Butterworth) and a 50 *Hz* notch filter (2^nd^ order IIR). After importing the filtered data into EEGLAB, an extended Infomax Independent Component Analysis (ICA) was applied for each participant (Delorme et al., 2007). Through visual inspection of the component scalp topography, time series, and power spectra, eye blink and lateral eye movement components were identified and removed from the data. The number of removed components for each participant is presented in Supplementary Table S4. Artefact-corrected data were exported back in Matlab format, and only data from Fz, Cz, and Pz electrodes were considered for further analysis.

Data were then analysed in the frequency domain using the Matlab *pspectrum* function. This function computes power spectra via FFT and employs Welch’s method to enhance the reliability of spectral estimates. By default, it segments the signal into as long sections as possible while ensuring a number of segments as close to (but not exceeding) 8, an overlap of 50%, and applying a Hamming window before computing the FFT. The averaged power spectra provided the final spectral estimates. To promote normality in data distribution, power values for each participant and trial were transformed to *dB*. Finally, the spectral power of theta (4–8 *Hz*), alpha (8–12 *Hz*), SMR (12–15 *Hz*), and beta (15–30 *Hz*) frequency bands was obtained by averaging the power estimates within the corresponding range.

### 2.7 Previous double-blind sham-controlled study

Maaz, Waroquier, et al. (2026) subjected 60 participants to a double-blind sham-controlled EEG-NF protocol. Participants were randomly allocated to either an Alpha-Up (*n* = 30) or a Sham (*n* = 30) EEG-NF session aiming at enhancing alpha power from parietal electrode. Both sessions were highly similar to the current Alpha-Down session. The session was also divided into training and transfer phases. During both phases, participants underwent through the exact, same procedure, with the same material, and received identical instructions. During the training phase, the same grey circle was continuously updated either from the participant’s real-time alpha power at Pz (Alpha-Up group) or from alpha trajectories prerecorded during an independent study (Sham group; Maaz, Paban, et al., 2026). Neither participants nor experimenters were informed about the veracity of the feedback and both groups were instructed to learn to maximise the circle size during training. Importantly, unlike the current Alpha-Down group, the circle size presented to the Alpha-Up group was positively proportional (*vs*. *inversely here*) to the participant’s alpha power. Therefore, the only difference between the current Alpha-Down group and the previous Alpha-Up and Sham groups was the targeted direction of alpha regulation, that is, down *vs*. up, respectively. Here, to generalise Maaz, Waroquier, et al. (2026) previous results to alpha downregulation, we systematically compared alpha trajectories from the current Alpha-Down group to the previous Alpha-Up and Sham groups.

### 2.8 Statistical analyses and hypotheses testing

#### 2.8.1 EEG data

For statistical analysis, the spectral power of theta, alpha, SMR, and beta frequency bands measured at Fz, Cz, and Pz electrodes was z-score standardised across all participants, regardless of their group. Alpha power at Fz, Cz, and Pz was defined as main outcome of interest. Analyses of remaining variables aimed to assess the neurophysiological specificity of the current EEG-NF session, that is, its ability to modulate specifically alpha power. For the purpose of statistical analyses, we arbitrarily hypothesised the presence of effects of interest on all variables. For analyses on training blocks, it implies that their spectral power is influenced by (i) trial repetition, (ii) the frequency of feedback update (5 *Hz vs*. 1 *Hz*, and 10 *Hz vs*. 5 *Hz*), (iii) the targeted direction of alpha regulation (Alpha-Up *vs*. Alpha-Down), (iv) the veracity of the feedback (Alpha-Down *vs.* Sham), and (v) each corresponding interaction effect. When considering the transfer block, their spectral power would be influenced by (i) trial repetition, (ii) the targeted direction of alpha regulation, (iii) the veracity of the feedback, and (iv) each corresponding interaction effect. The person who performed all statistical analyses (JM) remained blind to group assignments throughout.

Statistical analyses were conducted using Bayesian linear multilevel models implemented via the brms (Bürkner, 2017) and rstan (Stan Development Team, 2024) packages in R version 4.3.3 (R Core Team, 2024). Bayesians methods offer several advantages over traditional frequentist approaches (see Schad et al., 2021, 2023 for extended tutorials). This includes robustness in low-power contexts (Schönbrodt & Wagenmakers, 2018), straightforward implementation and computation of multilevel structures (Gelman et al., 2014), the ability to quantify evidence for both the presence and absence of effects (Dienes & Mclatchie, 2018), and reduced susceptibility to multiple comparison issues (Gelman et al., 2012). Each model treated one of the dependent variables as continuous and included the maximal random effect structure to account for interindividual variability (Barr et al., 2013). When analysing training blocks, the models included as fixed effects: the Trial number continuous within-subject predictor (i.e., integers from 1 to 8), the Frequency categorical within-subject predictor (i.e., 1 *Hz*, 5 *Hz*, and 10 *Hz*), and one Task categorical between-subject predictor (i.e., Alpha-Up, Alpha-Down, and Sham EEG-NF groups), as well as their interactions. The first trial served as reference level for the Trial predictor. Concerning Frequency and Task predictors, we applied repeated contrast matrixes following the guidelines of Schad et al. (2020), respectively presented in Supplementary Tables S5 and S6. Specifically, assigning a repeated contrast matrix to Task predictor enabled to evaluate both effects of the targeted direction of alpha modulation (Alpha-Up *vs*. Alpha-Down) and of the veracity of feedback (Alpha-Down *vs*. Sham). To prevent overfitting and constrain parameter estimates to plausible values, regularising priors of *N*(0, 1) were used for all model parameters (Schad et al., 2021). The full model equation (in brms syntax) was the following:

$$\begin{array}{l} Power\sim Trial\text{*}Frequency\text{*}Task\\ \;+\text{(}Trial\text{*}Frequency\text{ | }Subject). \end{array}$$ (1)

To analyse the transfer block, the same procedure was used while solely including the Trial and Task predictors, as well as their interaction as fixed effects. The corresponding model equation was defined as

Power~Trial*Task+(Trial | Subject). (2)

For all effects, Bayes Factors (BFs) quantified the strength of evidence in favour of one hypothesis over another (Dienes & Mclatchie, 2018). To ensure the stability of BFs, all reported analyses were performed twice (Schad et al., 2023). For each effect, the mean of the two obtained posterior distributions, as well as the largest limits of the 95% Credible Intervals (CrI), is reported. Additionally, we report the mean of the *BF_10_* indicating the strength of evidence for the alternative hypothesis (presence of an effect) relative to the null (absence of effect). Following Jeffreys (1939), we interpret *BF_10_* as follows: values above 3 indicate substantial evidence for the alternative hypothesis, values below ⅓ quantify substantial evidence for the null hypothesis, and values between ⅓ and 3 suggest data insensitivity to distinguish between the two hypotheses. When the presence of an effect is supported (i.e., *BF_10_* > 3), the *BF_10+_*, quantifying the amount of evidence for a positive-directional (i.e., one-sided) effect, is also reported. Supplementary Tables S8 and S9 present the model estimates (in standardised units), corresponding 95% CrI and BFs from all reported analyses.

#### 2.8.2 Feeling of feedback control and feedback credibility

To examine group differences in subjective experience of control and perceived feedback credibility, we conducted two Bayesian between-subject ANOVAs in R version 4.3.3 (R Core Team, 2024). The first test evaluated participants’ ratings of perceived control over the feedback variations, and the second assessed the belief that the feedback updates were random (corresponding, respectively, to questions (i) and (ii) in Material and neurofeedback implementation). Both variables were treated as continuous based on responses to 5-point Likert scale items ranging from 1—“Not at all” to 5—“Completely”. For each test, we report the estimated group difference along with the 95% CrI limits. In addition, we report the *BF_01_* which quantifies the strength of evidence in favour of the null (no group difference) over the alternative hypothesis (presence of group difference).

## 3 Results

### 3.1 Alpha increases no matter what during training

**Fig. 2.**
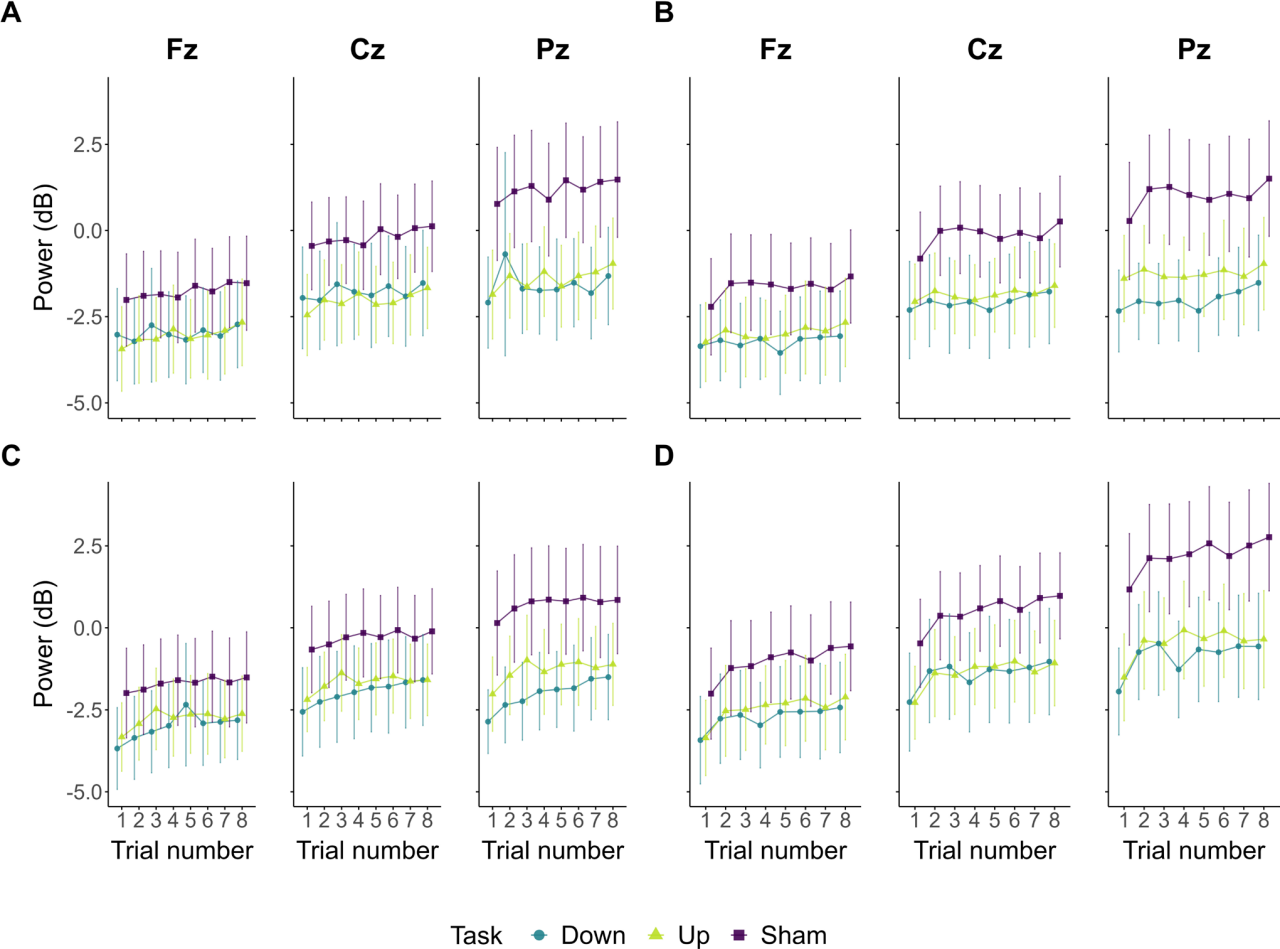
Alpha power evolution during each block of the neurofeedback session. Alpha power evolution across the trials of the training (A: 1 *Hz*; B: 5 *Hz*; C: 10 *Hz*) and transfer (D) blocks depending on electrode position (left: Fz; middle: Cz; right: Pz) and on the Task participants were subjected to (blue: Alpha-Down EEG-NF; green: Alpha-Up EEG-NF; violet: Sham EEG-NF). Each line point corresponds to EEG power averaged at the group level. Error bars indicate 95% confidence intervals.

Figure 2A–C shows alpha power evolution at frontal, central, and parietal electrodes during training depending on the Task to which participants were subjected (Alpha-Down EEG-NF, Alpha-Up EEG-NF, or Sham EEG-NF) and on the Frequency of feedback update (i.e., 1 *Hz*, 5 *Hz*, or 10 *Hz*). Globally, alpha power exhibits an upward trend during training independently of the task at hand and of the frequency at which the feedback was updated. This trend was confirmed by statistical analysis. Table 1 presents model estimates, 95% CrI, and corresponding BFs for the main effects of interest on alpha power during training (i.e., trial repetition, Task, Frequency of feedback update, and interactions between trial repetition and both Task and Frequency of feedback update).

**Table 1.** Main statistical results obtained on alpha spectral power during training.

Electrode	Parameter	Estimate	Lower	Upper	*BF_10_*	*BF_10+_*
**Fz**	**Trial**	**0.018**	**0.012**	**0.024**	**> 100**	**> 100**
Fz	Frequency - 5 Hz *vs*. 1 Hz	0.005	-0.067	0.078	0.037	1.254
Fz	Frequency - 10 Hz *vs.* 5 Hz	0.005	-0.061	0.07	0.033	1.248
Fz	Task - Up *vs.* Down	-0.024	-0.5	0.454	0.24	0.85
Fz	Task - Down *vs.* Sham	0.368	-0.104	0.844	0.796	15.309
Fz	Trial:Frequency - 5 Hz *vs.* 1 Hz	-0.003	-0.015	0.009	0.007	0.453
Fz	Trial:Frequency - 10 Hz *vs.* 5 Hz	0.009	-0.003	0.021	0.017	12.614
Fz	Trial:Task - Up *vs.* Down	-0.002	-0.017	0.012	0.008	0.597
Fz	Trial:Task - Down *vs.* Sham	0.001	-0.013	0.016	0.007	1.32
**Cz**	**Trial**	**0.019**	**0.013**	**0.025**	**> 100**	**> 100**
Cz	Frequency - 5 Hz *vs.* 1 Hz	0	-0.072	0.072	0.036	1.022
Cz	Frequency - 10 Hz *vs.* 5 Hz	-0.015	-0.088	0.057	0.04	0.518
Cz	Task - Up *vs.* Down	-0.028	-0.485	0.434	0.236	0.823
Cz	Task - Down *vs.* Sham	0.463	0.003	0.926	1.6	40.082
Cz	Trial:Frequency - 5 Hz *vs.* 1 Hz	-0.002	-0.013	0.009	0.006	0.552
Cz	Trial:Frequency - 10 Hz *vs.* 5 Hz	0.008	-0.005	0.02	0.013	7.607
Cz	Trial:Task - Up *vs.* Down	0.005	-0.01	0.02	0.01	2.837
Cz	Trial:Task - Down *vs.* Sham	-0.001	-0.017	0.014	0.008	0.754
**Pz**	**Trial**	**0.019**	**0.012**	**0.026**	**> 100**	**> 100**
Pz	Frequency - 5 Hz *vs.* 1 Hz	-0.048	-0.137	0.042	0.078	0.174
Pz	Frequency - 10 Hz *vs.* 5 Hz	-0.069	-0.144	0.008	0.19	0.04
Pz	Task - Up *vs.* Down	-0.132	-0.568	0.299	0.262	0.377
**Pz**	**Task - Down *vs.* Sham**	**0.671**	**0.244**	**1.103**	**25.434**	**> 100**
Pz	Trial:Frequency - 5 Hz *vs.* 1 Hz	0.002	-0.012	0.017	0.008	1.688
Pz	Trial:Frequency - 10 Hz *vs.* 5 Hz	0.011	-0.002	0.025	0.027	19.648
Pz	Trial:Task - Up *vs.* Down	0.005	-0.013	0.022	0.01	2.449
Pz	Trial:Task - Down *vs.* Sham	-0.003	-0.021	0.014	0.009	0.554

Each reported model has been computed twice to ensure the stability of the BFs. If not specified, each numerical value corresponds to the average of the values obtained across these two model computations. The “Estimate” column stands for the estimated group-level effects (slopes) of each model “Parameter” (in z-score standardised units). For the “Trial” predictor, the estimate corresponds to the group-level effect of one trial of the 1 *Hz* training block (defined as reference for subsequent comparisons for the Frequency predictor) of the Alpha-Up group (defined as reference for subsequent comparisons for the Task predictor). For the “Frequency” predictor, each comparison (i.e., “5 *Hz vs.* 1 *Hz*” and “10 *Hz vs.* 5 *Hz*”) estimate refers to the group-level effect during each training block on the first trial (modality of Trial predictor defined as reference for subsequent comparisons) of the Alpha-Up group. For the “Task” predictor, the estimate of both comparisons (“Alpha-Up *vs.* Alpha-Down” and “Alpha-Down *vs.* Sham”) refers to the between-group effect within the first trial of the 1 *Hz* training block. The “Lower” and “Upper” columns correspond to the minimal lower and maximal upper bounds of the two 95% CrI computed. The “*BF_10_*” and “*BF_10+_*” columns correspond to the BF in favour of the alternative hypothesis (relative to the null) and the directional (i.e., one-sided) BF, respectively.

Lines in bold highlight the effects for which BFs quantify sufficient evidence in favour of the alternative hypothesis over the null (i.e., presence of an effect).

Concerning the trial repetition effect, extreme evidence was quantified in favour of a positive effect on alpha power at Fz, Cz, and Pz (all *BF_10_* and *BF_10_*_+_ > 100; see Table 1). Regarding absolute power difference (i.e., Task predictors), substantial evidence supported the absence of difference between the Alpha-Up and Alpha-Down groups at Fz, Cz, and Pz (all *BF_10_* < $\frac{1}{3}$). Concerning the comparison between the Alpha-Down and Sham groups, *BF_10_* were insensitive towards neither the null nor the alternative hypothesis for an absolute power difference at Fz (*BF_10_* = 0.796) and Cz (*BF_10_* = 1.6). Yet, strong evidence favoured greater alpha power in the Sham than the Alpha-Down group at Pz (*BF_10_* = 25.434; *BF_10+_* > 100). Importantly, at Fz, Cz, and Pz, very strong to extreme evidence was found for the absence of interaction between trial repetition and both the targeted direction of alpha modulation (i.e., comparing alpha trajectories between Alpha-Up and Alpha-Down groups; all *BF_10_* < $\frac{1}{30}$ ) and feedback veracity (i.e., comparing alpha trajectories between Alpha-Down and Sham groups; all *BF_10_* < $\frac{1}{30}$ ). These results indicate that alpha power increased identically across groups (Alpha-Up, Alpha-Down, and Sham groups) during training.

Finally, regarding the effect of the frequency of feedback update and remaining interactions, we mostly found substantial to extreme evidence supporting the absence of an effect on alpha power (all *BF_10_* < $\frac{1}{3}$). The only exception, for which we found insensitive evidence, was the interaction between the frequency of feedback update (5 *Hz vs*. 1 *Hz*) and the targeted direction of alpha modulation (up *vs*. down) on alpha power at Cz and Pz. All model estimates, 95% CrI, and corresponding BFs are reported in Supplementary Table S7. Overall, these results suggest that alpha power tends to increase within an EEG-NF session, yet independently of the feedback update frequency, the veracity of feedback, or the targeted direction of alpha modulation.

### 3.2 Alpha non-stationarity is preserved during the transfer phase

The evolution of alpha power during the transfer block is presented in Figure 2D as a function of participants’ group (Alpha-Down, Alpha-Up, Sham). As during training, alpha power exhibits an increase across the transfer trials irrespective of group allocation. These results were confirmed statistically. Extreme evidence supported a positive effect of trial repetition on alpha power at Fz, Cz, and Pz (all *BF_10_* and *BF_10+_* > 100; see Table 1). Concerning absolute group difference in alpha power between the Alpha-Down and Alpha-up groups, substantial evidence supported the absence of group difference at Fz, Cz, and Pz (all *BF_10_* < $\frac{1}{3}$). When comparing the Alpha-Down and the Sham groups, insensitive evidence was quantified at Fz (*BF_10_* = 0.928) and Cz (*BF_10_* = 1.268), while, at Pz, strong evidence was found in support of superior absolute alpha power in the Sham compared with the Alpha-Down group (*BF_10_* = 10.479; *BF_10+_* > 100). Finally, strong to very strong evidence was found in favour of the absence of interaction between trial repetition and both the targeted direction of alpha modulation (i.e., comparing alpha trajectories between Alpha-Down and Alpha-Up groups) and the veracity of feedback (i.e., comparing alpha trajectories between Alpha-Down and Sham groups; all *BF_10_* < $\frac{1}{10}$ ). All model estimates, corresponding 95% CrI and BFs, are reported in Table 2. These results suggest that alpha power continues to increase during the transfer phase regardless of the targeted direction of alpha modulation and of the veracity of feedback.

**Table 2.** Estimates from models computed on alpha spectral power during the transfer block.

Electrode	Parameter	Estimate	Lower	Upper	*BF_10_*	*BF_10+_*
**Fz**	**Trial**	**0.037**	**0.028**	**0.047**	**> 100**	**> 100**
Fz	Task - Up *vs*. Down	-0.044	-0.542	0.449	0.255	0.753
Fz	Task - Down *vs*. Sham	0.402	-0.1	0.896	0.928	17.172
Fz	Trial:Task - Up *vs*. Down	-0.005	-0.028	0.018	0.013	0.492
Fz	Trial:Task - Down *vs*. Sham	0.017	-0.006	0.04	0.034	12.616
**Cz**	**Trial**	**0.036**	**0.026**	**0.046**	**> 100**	**> 100**
Cz	Task - Up *vs*. Down	0.01	-0.481	0.502	0.25	1.062
Cz	Task - Down *vs*. Sham	0.452	-0.047	0.947	1.268	26.42
Cz	Trial:Task - Up *vs*. Down	-0.002	-0.026	0.022	0.012	0.732
Cz	Trial:Task - Down *vs*. Sham	0.015	-0.009	0.039	0.026	8.218
**Pz**	**Trial**	**0.032**	**0.022**	**0.042**	**> 100**	**> 100**
Pz	Task - Up *vs*. Down	-0.09	-0.569	0.392	0.262	0.55
**Pz**	**Task - Down *vs*. Sham**	**0.673**	**0.19**	**1.151**	**10.479**	**> 100**
Pz	Trial:Task - Up *vs*. Down	0.004	-0.021	0.029	0.013	1.655
Pz	Trial:Task - Down *vs*. Sham	0.01	-0.015	0.035	0.017	3.569

Each reported model has been computed twice to ensure the stability of the BFs. If not specified, each numerical value corresponds to the average of the values obtained across these two model computations. The “Estimate” column stands for the estimated group-level effects (slopes) of each model “Parameter” (in z-score standardised units). For the “Trial” predictor, the estimate corresponds to the group-level effect of one trial within the Alpha-Up group (defined as reference for subsequent comparisons for the Task predictor). For the “Task” predictor, the estimate of both comparisons (“Up *vs.* Down” and “Down *vs.* Sham”) refers to the between-group effect within the first trial of the transfer block (defined as reference for subsequent comparisons for the Trial predictor). The “Lower” and “Upper” columns correspond to the minimal lower and maximal upper bounds of the two 95% CrI computed. The “*BF_10_*” and “*BF_10+_*” columns correspond to the BF in favour of the alternative hypothesis (relative to the null) and the directional (i.e., one-sided) BF, respectively.

Lines in bold highlight corresponding electrodes and parameters for which BFs quantify sufficient evidence in favour of the alternative hypothesis over the null (i.e., presence of an effect) on alpha power.

### 3.3 Neurophysiological specificity: side effects on theta and SMR

**Fig. 3.**
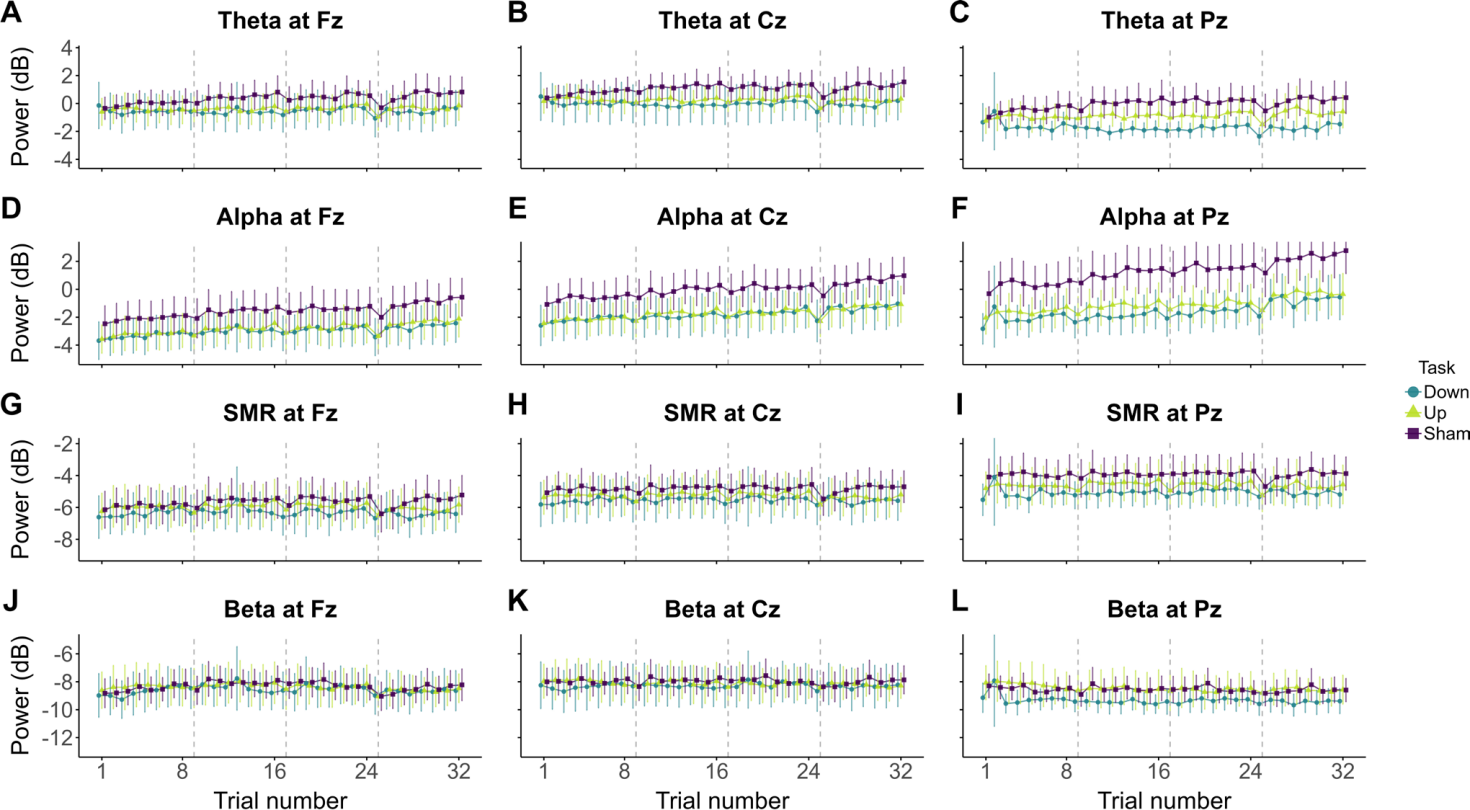
Evolution of EEG spectral power throughout a single EEG-NF session. Theta (4–8 *Hz*; A–C), alpha (8–12 *Hz*; D–F), SMR (12–15 *Hz*; G–I), and beta (15–30 *Hz*; J–L) power evolution over the entire EEG-NF session, including training and transfer phases. Each line point represents the EEG spectral power at one trial. Each was averaged at the group level in function of the Task participants were subjected to (blue: Alpha-Down EEG-NF; green: Alpha-Up EEG-NF; violet: Sham EEG-NF). Error bars indicate 95% confidence intervals. Vertical dashed lines mark the first trial of each new block.

To evaluate the neurophysiological specificity of the current alpha EEG-NF protocol (i.e., its ability to specifically downregulate alpha power solely), we reproduced the above-mentioned analyses on the spectral power of theta (4–8 *Hz*), SMR (12–15 *Hz*), and beta (15–30 *Hz*) frequency bands. Depending on participants’ group (Alpha-Down, Alpha-Up, or Sham), Figure 3 displays power evolution within each considered band throughout the EEG-NF session. For simplicity, all model estimates and corresponding 95% CrI and BFs are reported in Supplementary Tables S8 and S9, respectively, regarding the analyses of the training and the transfer phases.

Concerning training blocks, we found substantial to very strong evidence for an effect of the frequency of feedback update (10 *Hz vs*. 5 *Hz* with a superiority in power for the 5* Hz* frequency) on theta power (Fz: *BF_10_* = 4.304, *BF_10+_* = 0.001; Cz: *BF_10_* = 38.64, *BF_10+_* ≈ 0; Pz: *BF_10_* = 70.53, *BF_10+_* ≈ 0). For all remaining considered effects, insensitive or substantial to extreme evidence for an absence of effect was quantified. This indicates that visual updating of the feedback at 5 *Hz* elicits a constant increase in theta power over time regardless of the task at hand (i.e., entrainment effect of visual feedback; see Supplementary Figs. S1 and S2).

Regarding the transfer phase, we found strong to extreme evidence for a positive effect of trial repetition on theta power at all electrodes and on SMR power at Fz and Cz (all *BF_10_* and *BF_10+_* > 10). Additionally, substantial evidence was found regarding absolute difference in theta power at Pz between the Alpha-Down and the Sham groups (favouring a superiority in the Sham group: *BF_10_* = 7.065, *BF_10+_* > 100). For all remaining considered effects, either insensitive or substantial to extreme evidence for no effect was quantified. These results suggest that alpha neighbouring bands, that is, theta and SMR, may also exhibit a positive non-stationarity over time no matter the group.

### 3.4 Neurofeedback is perceived similarly between groups

**Fig. 4.**
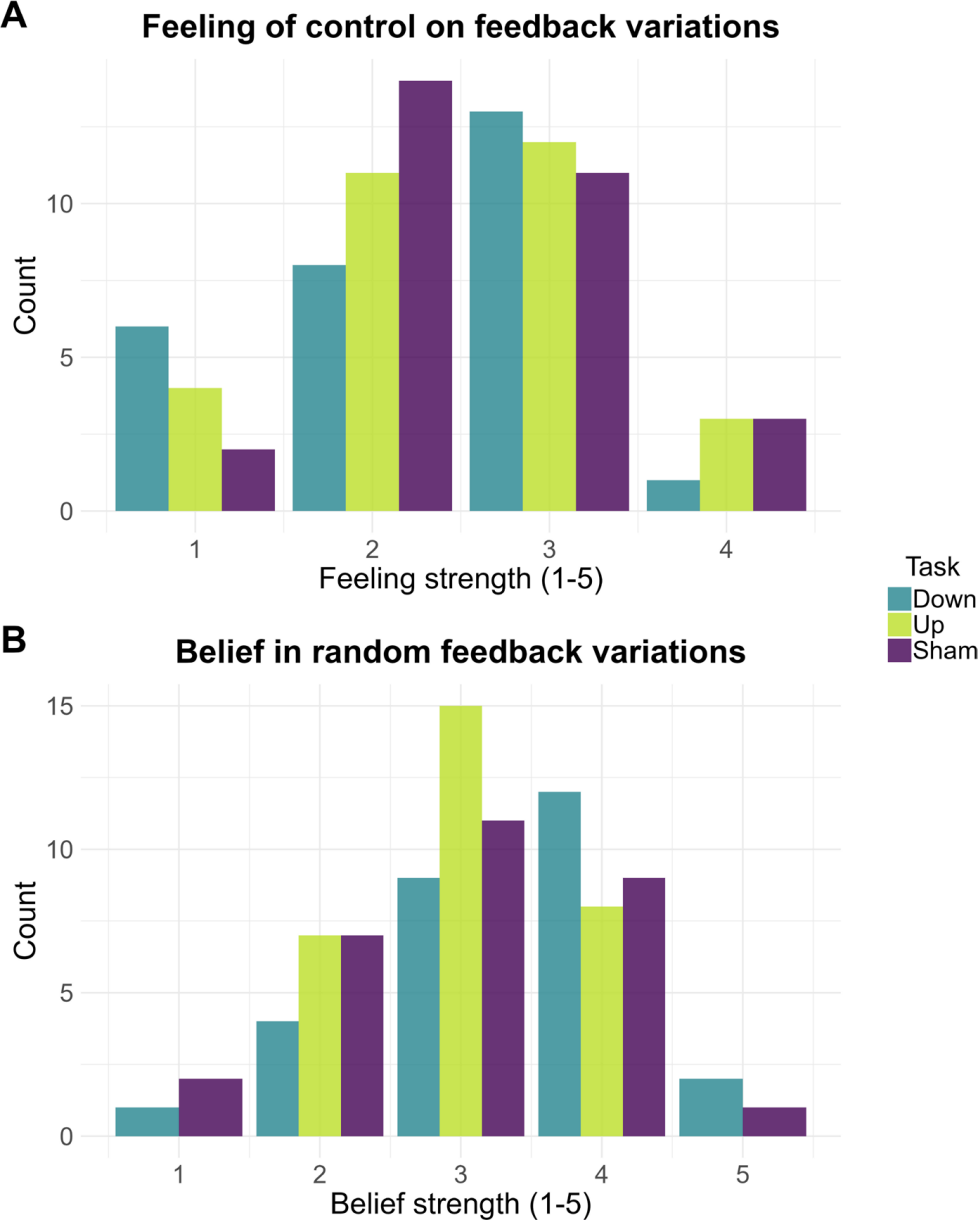
Distributions of responses on participants’ feeling of control and belief in random feedback between groups. Distribution of ratings (5-point Likert scale) of participants’ feeling of control over feedback variations (A) and belief in the random (i.e., sham) nature of feedback variations (B), depending on whether participants were subjected to an Alpha-Down (blue), Alpha-Up (green), or Sham (violet) EEG-NF session.

Finally, we evaluated whether participants underwent a similar experience of the feedback in all three groups (Alpha-Down, Alpha-Up, and Sham). Figure 4 presents the distribution of the strength of participants’ feeling of control over feedback variations (Fig. 4A), and of participants’ belief that feedback variations were actually random and not based on their EEG activity (i.e., sham; Fig. 4B). Bayesian independent ANOVAs quantified substantial evidence for an absence of group difference in both responses (Feeling of control: β = -0.082, 95% CrI [-0.308, 0.137], *BF_01_* = 7.370; Belief in random feedback variations: β = 0.181, 95% CrI [-0.055, 0.435], *BF_01_* = 3.282). Importantly, these suggest that participants experienced similar control over feedback variations and that feedback veracity (i.e., genuine *vs*. sham) could not have been guessed.

## 4 Discussion

This study rigorously evaluated whether alpha power could be downregulated during a single EEG-NF session. One group of healthy individuals was subjected to an EEG-NF training aiming to downregulate their alpha power at parietal electrode. Importantly, to control for non-specific influences, alpha trajectories from the Alpha-Down group were compared with independent Alpha-Up and Sham groups, respectively, subjected to genuine EEG-NF aiming alpha upregulation (*vs*. current downregulation) and to sham EEG-NF. During training, alpha power showed robust within-session increases at frontal, central, and parietal electrodes. Critically, these upward trajectories occurred irrespective of the targeted regulation direction (Alpha-Down vs. Alpha-Up), the veracity of the feedback (genuine vs. sham), and the frequency of feedback update. Additionally, the same pattern of results was observed during a transfer phase, following EEG-NF training, aiming to evaluate participant self-regulation skills. Theta and SMR bands also exhibited positive drifts, and subjective ratings indicated comparable perceived control and successful blinding across groups. Collectively, these findings argue against volitional alpha self-regulation under single-session EEG-NF settings and instead support a dominant role of non-specific, time-dependent factors.

Historically, EEG modulation through EEG-NF is considered to be underlined by an active self-regulation mechanism (e.g., Micoulaud-Franchi & Fovet, 2018). This mechanism is hypothesised to elicit, through volitional control, significant EEG changes within and between training sessions, as well as between EEG resting states measured before and after training (Ros et al., 2020). In particular, the alpha band (8–12 *Hz*) spectral densities have been identified as a privileged and easy-to-modulate EEG outcome (Belinskaia et al., 2020; Chikhi et al., 2023; Escolano et al., 2014; Kluetsch et al., 2014; Ros et al., 2010). Specifically, alpha downregulation has been targeted and achieved in single EEG-NF sessions (Nan et al., 2020; Ros et al., 2013). However, by contrast, this study did not achieve alpha downregulation through a single EEG-NF session. Rather, we observed extreme Bayesian evidence for an increase in alpha power during both training and transfer phases (Fig. 2).

These results call for a careful reappraisal of earlier reports of alpha downregulation in single EEG-NF sessions (Kluetsch et al., 2014; Nan et al., 2020; Ros et al., 2010, 2013). Crucially, the current contrasting results may be explained by several methodological limitations present in previous designs. For example, Ros et al. (2013) and Kluetsch et al. (2014) interpreted successful alpha downregulation based on comparisons of average power during the entire training session with resting-state baselines taken beforehand. However, such a comparison does not reveal whether alpha decreased as training progressed. The observed decrease could be explained by the simple engagement in the EEG-NF task, which may tend to suppress alpha relative to rest because of attentional demands (Jensen, 2024). More importantly, these previous studies did not systematically include controls for non-specific EEG changes. In particular, sham EEG-NF represents the most common control condition for non-specific influences on EEG-NF outcomes (Sorger et al., 2019). Participants are engaged in a similar genuine EEG-NF procedure, yet they receive feedback disconnected from their EEG activity. Sham was implemented twice in alpha downregulation studies (Nan et al., 2020; Ros et al., 2013). In those cases, authors confirmed superiority in alpha modulation for genuine EEG-NF over sham. However, these results were limited by very low sample sizes which are known to inflate the rate of type I errors (Button et al., 2013). Consequently, the possibility to downregulate alpha activity during single EEG-NF sessions remained to be determined by further studies with proper controls and sufficient sample sizes.

In addition to sham, bidirectional control groups have emerged as particularly powerful alternatives (Sorger et al., 2019). While accounting for non-specific influences, participants are trained to either increase or decrease the same EEG feature, evaluating the specificity of the targeted direction of EEG modulation. Building on previous methodological issues, the current study implemented both sham and bidirectional controls by systematically comparing alpha trajectories of the Alpha-Down group with two independent groups from a previous study (Maaz, Waroquier, et al., 2026). Importantly, we reproduced identical material, procedure, and instructions from previous settings. The previous double-blind sham-controlled study investigated the mechanisms of alpha upregulation during a single EEG-NF session. One group underwent an Alpha-Up session aiming alpha upregulation, whereas the other a Sham session. In previous work, alpha showed clear increases in power throughout the session independently of the veracity of feedback (i.e., Alpha-Up *vs*. Sham). These findings suggested that single-session alpha upregulation was not actually driven by volitional EEG control claimed by previous studies (e.g., Agnoli et al., 2018; Chikhi et al., 2023).

Here, we aimed to extend previous conclusions on alpha power upregulation to its downregulation in single-session designs. Comparing the current Alpha-Down with previous Alpha-Up and Sham groups enabled to robustly evaluate whether alpha downregulation is affected by the targeted direction of alpha modulation (Alpha-Down *vs*. Alpha-Up) and by feedback veracity (Alpha-Down *vs*. Sham; Sorger et al., 2019). In terms of absolute between-group differences during training and transfer, Bayesian analyses supported similar levels of alpha power between the Alpha-Down and the Alpha-Up groups, yet favoured superior absolute alpha power in the Sham compared with the Alpha-Down group at Pz (insensitive evidence at Fz and Cz; see Table 1). This is consistent with a previous report of superior alpha amplitude in a sham compared with a genuine EEG-NF group aiming alpha downregulation (yet with no interaction between feedback veracity and time; Nicholson et al., 2023). More importantly, during both training and transfer phases, Bayesian evidence strongly supported identical alpha increases between all three groups (Tables 1 and 2). The absence of interaction with the targeted direction of alpha modulation and feedback veracity indicates that alpha activity may not be effectively downregulated through EEG-NF, but is rather rising due to non-specific influences. In addition, when evaluating subjective experience of control over feedback and its credibility, results confirmed no difference between all three groups (Fig. 4). Together, these results do not support the historical claim of volitional EEG control (e.g., Strehl, 2014). Instead, they are consistent with the view that alpha power non-specifically increases during an EEG-NF session. In particular, this increase occurs independently of the targeted direction of alpha modulation, the veracity of feedback, the initial alpha power values (e.g., Nicholson et al., 2023), and whether participants are provided or not with a real-time feedback.

One could argue that the current EEG-NF paradigm may not optimally engage participants in the targeted self-regulation task. Successful EEG modulation through EEG-NF is generally assumed to depend on sustained cognitive engagement in the self-regulation task (Micoulaud-Franchi et al., 2015). Yet, the present study did not incorporate measures of motivation, engagement, or task-related effort during training. This thus limits our ability to ensure participants’ engagement throughout the session. Relatedly, some have argued that more immersive or gamified EEG-NF environments may enhance motivation and engagement, thereby potentially facilitating specific EEG self-regulation (Gaume et al., 2016; Wood et al., 2014). However, to the best of our knowledge, this possibility remains to be determined by further studies combining such gamified paradigms (e.g., Chang et al., 2022; Wang et al., 2025) with rigorous control conditions, as implemented here, along with motivation and engagement measures.

Moreover, the present findings on alpha power are consistent with prior EEG-NF studies documenting alpha increases without alpha possibility to be modulated (Belinskaia et al., 2020; Chikhi et al., 2024; Dessy et al., 2020; Jiang et al., 2021; Naas et al., 2019). For instance, alpha power increases were similarly present in a sham and a genuine EEG-NF protocols (Naas et al., 2019), during a theta (4–8 *Hz*) EEG-NF session (Chikhi et al., 2024), and within and between the sessions of a biofeedback protocol targeting heart rate variability (Dessy et al., 2020). In addition, recent evidence demonstrated that alpha similarly increases when participants are exposed to a passive visualisation task mimicking the environment of an EEG-NF session (Maaz et al., 2025; Maaz, Paban, et al., 2026; Maaz, Waroquier, et al., 2026). Overall, it seems that alpha power tends to increase over time independently of participant engagement in EEG-NF.

Outside of EEG-NF settings, it is already well documented that alpha activity is exposed to significant non-stationarities occurring over milliseconds to hours (e.g., Benwell et al., 2019; Cohen, 2014; Kopčanová et al., 2025). Namely, irrespective of any experimental manipulation, alpha power tends to increase over time, whereas alpha peak frequency tends to decrease. These non-stationarities can be explained by time-on-task documented effects on variables such as fatigue, arousal fluctuations, and reduced vigilance, which are known to influence alpha activity (Eastwood et al., 2012; Maaz, Waroquier, et al., 2026). For example, cognitive fatigue increases with time-on-task and has been linked to an increase in alpha power (Käthner et al., 2014). Similarly, alpha power increases over time have been associated with an increased occurrence of mind wandering (i.e., shifts in attentional focus towards internal, task-unrelated thoughts; Arnau et al., 2020; Compton et al., 2019; Seli et al., 2016). These time-dependent factors are inherently present in EEG-NF sessions (whether genuine or sham). However, while these variables may reasonably underlie the current non-specific increases in alpha power, this study lacked proper measures to specifically identify the time-dependent factor(s) responsible for the time-on-task effects on alpha power. This remains to be determined by further EEG-NF studies incorporating additional measures of such time-dependent factors. These could include the use of “thought probes” to evaluate mind-wandering occurrence during EEG-NF (e.g., Arnau et al., 2020), or continuous measurement of pupil size to track potential vigilance decrement (e.g., Martin et al., 2022). Still, the current findings are congruent with the idea that, no matter what, alpha power exhibits positive non-stationarities due to general time-on-task effects (see Fig. 3D–F showing alpha rising throughout the experimental session irrespective of participant’s group). They enable to generalise previous conclusions on alpha upregulation to its downregulation, and call for caution in interpreting alpha EEG-NF results without proper systematic controls.

Furthermore, we would like to stress the relevance of the observed results during the transfer phase. Typically, transfer phases are recommended to evaluate the assumption that participants, after sufficient training, should become able to self-regulate their EEG activity without the help of the real-time feedback (Ros et al., 2020; Strehl, 2014). However, their implementation remains scarce in existing studies (for exceptions, see Paban et al., 2024; Strehl et al., 2006). Here, alpha power continued to rise during the no-feedback transfer phase irrespective of participant group (Fig. 2D), suggesting that no learned EEG modulation occurred during training. Additionally, we manipulated the frequency of feedback update during training (1, 5, or 10 *Hz*) to evaluate whether feedback update timing would influence learning of alpha modulation. However, as no successful EEG modulation was achieved, we congruently observed no effect (nor corresponding interactions) of feedback update frequency on alpha power. Thus, all observed results on alpha power converge towards a failure of its volitional modulation in the present single-session setting.

Another assumption in EEG-NF research is the neurophysiological specificity of training, that is, whether the EEG-NF protocol solely modulates the targeted EEG feature (Sorger et al., 2019). However, again, this assumption has still not been systematically addressed in previous studies. Here, we reproduced the analyses on alpha on the spectral power of theta, SMR, and beta frequency bands. The results of the training and transfer phases are presented, respectively, in Supplementary Tables S8 and S9. During training, we only obtained substantial evidence for a superior theta power when the feedback was updated at 5 *Hz* (*vs*. 10 *Hz*), which is probably due to an entrainment effect of the 5 *Hz* frequency (see Supplementary Figs. S1 and S2). Although we confirmed the absence of an effect on alpha, SMR, and beta power, this suggests that the continuous update of visual feedback may artificially modulate the EEG features (here theta power) targeted by EEG-NF.

Importantly, these results have implications for the design and interpretation of EEG-NF studies. Specifically, a common practice is to infer successful EEG modulation by comparing targeted EEG features during training to brief resting-state “baseline” measures taken beforehand. These so-called baselines are usually measured during the passive fixation on a cross (e.g., Agnoli et al., 2018; Dempster & Vernon, 2009; Gonçalves et al., 2018; Maszczyk et al., 2020; Ros et al., 2013). However, although this practice was recommended in the past (Ancoli & Kamiya, 1978), it is now considered as problematic (Strehl, 2014). The main reason for this is the assumption that any deviation of targeted EEG features from their baseline during training necessarily reflects specific, volitional EEG modulation. Rather, these baselines may also differ from EEG-NF training through participants’ active engagement in the self-regulation task (which may alter EEG spectral densities, especially of the alpha band; Jensen, 2024). In addition, during baseline measures, there is usually no stimulus continuous update as feedback, in contrast to training. As such, substantial differences in spectral densities between baseline and training may also reflect perceptual processing of feedback continuous updating (Maaz et al., 2025).

In particular, modifying visual stimuli at specific frequency rates may influence EEG spectral densities of the theta and/or alpha band (Spaak et al., 2014; VanRullen, 2016). During the current training phase, we consistently observed an increase in theta power entrained by a frequency of feedback update of 5 *Hz* (*vs*. 10 *Hz*; see Supplementary Figs. S1 and S2). This stimulus-driven, entrained theta increase calls for caution when interpreting the results of studies that relied on baseline measures, especially those targeting theta modulation (e.g., Egner & Gruzelier, 2004; Rozengurt et al., 2016). Furthermore, even without using baselines, the frequency used for continuous feedback update may additionally mask or saturate the dynamics of targeted features, limiting the potential for specific self-regulation during actual EEG-NF training. Therefore, we recommend future EEG-NF studies to assess for perceptual feedback-driven effects on the targeted features, or to use, for instance, an arrhythmic continuous update of feedback to prevent their occurrence.

Moreover, during the transfer phase, we interestingly observed an effect of trial repetition, with the absence of any corresponding interactions, on theta power at Fz, Cz, and Pz, as well as on SMR power at Fz and Cz (Fig. 3A–C, G–I). Bayesian analyses also quantified substantial evidence for a superiority of the Sham over the Alpha-Down group in terms of absolute theta power at Pz (similar to the above-mentioned findings on alpha power). Overall, these results suggest that the spontaneous, non-specific increases in alpha power may extend to its neighbouring theta and SMR bands. They are consistent with previous work documenting spontaneous theta and SMR increases over time in EEG-NF settings (Dessy et al., 2020; Grosselin et al., 2021; Maaz, Waroquier, et al., 2026; Schabus et al., 2017). The present multiband analyses, therefore, suggest that the observed changes may extend to global, broadband non-specific dynamics rather than band-specific, feedback-driven learning.

More broadly, EEG-NF research mostly focused on establishing effective clinical and behavioural interventions (Chiasson et al., 2023; Micoulaud-Franchi et al., 2015; Thibault et al., 2015). To this end, EEG-NF studies implemented single- or multiple-session trainings targeting the modulation of specific EEG features supposed to underly clinical and behavioural outcomes. However, despite the fact that EEG modulation remains the first objective of EEG-NF, EEG outcomes (within-, between-session changes, and pre- to post-training changes during rest) have for long been ignored and implicitly assumed (Thibault & Raz, 2018). As a result, the core assumption that EEG changes occur through volitional control (i.e., active self-regulation mechanism) has been poorly evaluated (Micoulaud-Franchi & Fovet, 2018). Specifically, the present work rigorously addressed the case of claimed alpha modulation during single EEG-NF sessions (Agnoli et al., 2018; Chikhi et al., 2023; Nan et al., 2020; Ros et al., 2013). The results suggest that neither the up- nor downregulation of alpha power occurs through the hypothesised volitional control. Rather, they are congruent with documented spontaneous alpha non-stationarities over time (e.g., Benwell et al., 2019). This emphasises the importance for further research to implement systematic control of non-specific influences on EEG-NF outcomes.

Lastly, as the present study evaluated alpha modulation within a single session, one could argue that its conclusions only apply to within-session EEG changes of alpha EEG-NF. Yet, previous studies also reported non-specific alpha increases between sessions (Dessy et al., 2020; Naas et al., 2019), as well as between resting-state measures (Chikhi et al., 2024; Ros et al., 2013). Similarly, the current increases in theta and SMR power highlight that non-specific broadband changes may occur during EEG-NF. Following current recommendations on brain and behavioural outcomes, this study emphasises the necessity to properly assess the specificity of targeted EEG modulation (Ros et al., 2020). Indeed, we do not claim that this study rules out the possibility to specifically modulate EEG activity through EEG-NF. EEG-NF studies differ significantly in their protocol design, even among protocols targeting the same EEG features (Chiasson et al., 2023; Hasslinger et al., 2022). Hopefully, particular settings, different from the current EEG-NF session, might enhance the probability of observing specific EEG modulation. However, ensuring this specificity is still not a standard practice in the field. Guidelines on the justification and the choice of control conditions for neurofeedback already exist (Sorger et al., 2019). We thus recommend future studies to refer to these guidelines when conceptualising EEG-NF protocols.

Consequently, further research might be able to delineate non-specific from specific components of the targeted EEG modulation. Under such conditions, the non-specific components of EEG changes could be statistically modelled and separated to obtain clearer estimates of EEG-NF specific effects. Given the high between- (*vs*. within-) subject variability of EEG spectral densities (Kadosh & Staunton, 2019; Uudeberg et al., 2025), this approach may be particularly informative using a within-subject design, in which participants undergo multiple EEG-NF protocols (e.g., genuine *and* sham EEG-NF training). Such designs would allow direct comparison of modulation slopes within the same individual, thereby reducing between-subject variability and facilitating the separation of global non-specific drifts from EEG-NF specific changes in EEG activity.

## 5 Conclusion

Under matched procedures and rigorous controls, single-session EEG-NF did not confer volitional control over alpha power. Instead, alpha power exhibited robust non-stationary increases over time that were insensitive to feedback veracity, targeted regulation direction, and feedback update frequency. These results, together with prior work, argue that the within-session modulation of classic EEG rhythms such as the alpha band cannot be attributed to feedback-specific learning in the absence of well-controlled evidence.

## Supplementary Material

Supplementary Material

## Data Availability

Supplementary Table S10 presents the checklist of the Consensus on the reporting and experimental design of clinical and cognitive-behavioural neurofeedback studies (CRED-nf). All materials, data, and analysis codes are available via the Open Science Framework: https://osf.io/6bn4v.
