# Supplementary material for: Non-specific increase in alpha power during a neurofeedback session targeting its downregulation
Source: Imaging Neurosci (Camb). 2026 May 29;4:IMAG.a.1258. doi: 10.1162/IMAG.a.1258 (PMC13224316; doi:10.1162/IMAG.a.1258)
Supplement: Supplementary Material [file IMAG.a.1258_supp.pdf]

Supplementary Material

for:

Non-specific increase in alpha power during a neurofeedback session targeting its downregulation

Jacob Maaz,[1,2,3] Alexandra Dia,[1,2] Laurent Waroquier,[4] Véronique Paban,[1,2] and Arnaud Rey[1,3]

Author affiliations:

1 Aix Marseille Univ, CNRS, CRPN, 13331 Marseille, France

2 Institute Neuro-Marseille, Aix Marseille Univ, France

3 Institute of Language Communication and the Brain, Aix Marseille Univ, France

4 Aix Marseille Univ, PSYCLE, 13621 Aix-en-Provence, France

Correspondence to: Jacob Maaz

Centre for Research in Psychology and Neuroscience (CRPN) – UMR 7077

CNRS – Aix-Marseille Université

3, place Victor Hugo – Case D

13331 Marseille Cedex 3 – France

jacob.maaz@univ-amu.fr

Supplementary Table 1 Verbal instructions provided to participants before the training and the transfer phases.

Phase	Verbal instructions (translated from French)
**Training**	You will complete three blocks of neurofeedback training. Each block is composed of eight one-minute trials. During the eight trials of a block, a circle will be presented at the centre of the screen while continuously growing and decreasing in size. Depending on the block, its size will grow and decrease at different rates. At any given moment, the size of the circle reflects the state of your brainwaves in real-time, which are influenced by your thoughts. By keeping your eyes on the circle, your task is thus to try your best to increase as much as possible the size of the circle thanks to your thoughts. The goal is to find the best mental strategy that effectively renders the circle as big as possible. Over the course of the trials, you will progressively become able to self-modulate your brainwaves by intentionally modifying your thoughts and adopting the best mental strategy.
**Transfer**	Congrats for completing this training! You will now complete a final block, but without feedback. For another eight one-minute trials, a circle will be presented at the centre of the screen. However, this time, the size of the circle will not be modified. By keeping your eyes on the circle, your task will still be to try your best to self-regulate your brainwaves thanks to your thoughts. The objective is to reproduce the mental strategies you used during the training blocks to increase the circle size. Keep it up, it’s almost done!

Supplementary Table 2 Checklist guidelines for time-frequency analyses (adapted from Keil et al., 2022).

#	Information to be included in the manuscript	Completed?
1	The specific stage of processing in which time-frequency analysis was applied (e.g., single-trials, after trial averaging, etc.). This clarifies which aspect(s) of oscillatory activity (e.g., spontaneous and/or induced, evoked, etc.) are being observed. If averaged potentials of each trial were subtracted prior to conducting time-frequency analyses on single trials, this step should be stated along with figures depicting the averaged potential in both time and frequency domains.	YES
2	For authors using Fourier-based time-frequency analyses (spectrograms), the following recommendations are provided for each specific approach: (1) If using spectrograms, or moving-window DFT/FFT analyses, report the specific window size and step size. Additional within-window averaging averaging achieved via algorithms (e.g., Welch periodogram method) should also be reported. (2) If using multitaper analyses, the type of tapering windows used, total number used, their center frequencies, whether any smoothing factors are applied, and the specific algorithms used to form their shapes should be reported. (3) If using complex demodulation, the frequencies examined, and the specific properties of the low-pass filter used (i.e., filter type, order, and cutoff frequency) should be reported.	YES
3	For authors conducting time-frequency analyses based on time domain filtering methods (i.e., Filter-Hilbert or similar approaches), the software and version number of the Hilbert transform used to identify the phase-shifted version of the empirical signal. In addition, authors should state the specific properties of band-pass filter (i.e., filter types, order, and cutoff frequencies).	Not applicable
4	If using wavelet-based methods for time-frequency analyses, include the smoothing/ smearing for the minimum and maximum frequency of interest and indicate the maximal temporal and frequency smoothing for a specific wavelet family. In addition, using Morlet wavelets, include the Morlet parameter (m) indicating the trade-off between time and frequency smoothing and smoothing values in the time (σt) and frequency (σf) domains.	Not applicable
5	As for frequency domain analyses, specify the duration of analytical time segments used, with pre- and post-event onset duration. In addition, include the number of time segments for each condition/group.	YES
6	Descriptions of any nonlinear transformations and/or baseline adjustment that were used prior to statistical analyses, accompanied by a rationale for these decisions. Specifically, include the duration used as a baseline and the type of algorithm (e.g., division, subtraction, etc.) used for this adjustment.	Not applicable

Supplementary Table 3 Checklist guidelines for spectral analyses (adapted from Keil et al., 2022).

#	Information to be included in the manuscript	Completed?
1	Specifying the inputs and outputs of all algorithms used in the processing pipeline	YES
2	A discussion of how oscillatory activity was conceptualised relative to 1/*f* noise and/or other broadband phenomena (underlying model)	YES
3	A rationale for the choice of measurement of power in a specific frequency band, including how nonperiodic (1/*f*) contributions to the spectrum were addressed	YES
4	A statement describing the specific type of Fourier- or non-Fourier-based algorithm used for transformation from the time domain to the frequency domain	YES
5	The exact duration of time segment used for transformation into the frequency domain for each condition of interest. In addition, the total number of segments (e.g., trials per condition) entering an averaged spectrum, along with how data epochs were combined within and across recordings (e.g., overlapping windows)	YES
6	The type, total number of, overlap between, and duration of any taper window functions, along with their ramp-on and ramp-off duration. If alternative and/or additional steps were taken to address edge artifacts, these should be stated. If applicable, the choice of taper window function should be specified as being guided by computational principles and/or by aiming to replicate current methods (e.g., Hann or Hamming window)	YES
7	If zero-padding is applied, the number and location of added zeros (e.g., before the time series, after the time series, or both before and after the time series)	Not applicable
8	All normalisation steps (e.g., by length of time, multiplication of the lower half of the spectrum, or by complex conjugate, etc.) applied to the spectral power or power density calculation	YES
9	The native frequency resolution of the spectrum (e.g., 1/(epoch duration in seconds)). In addition, the number of frequency bins extracted for a specific band of interest, and the range of these binds (e.g., 7.98 Hz to 11.97 Hz)	YES
10	Whether analyses were conducted using single trials or the average across trials	YES
11	How band power was measured from a spectrum	YES

Supplementary Table 4 Number of Independent Components removed from the data of each participant in the Alpha-Down group.

Participant's number	Number of Independent Components removed
1	2
2	3
3	2
4	3
5	3
6	2
7	2
8	2
9	2
10	2
11	2
12	3
13	3
14	2
15	3
16	3
17	3
18	2
19	3
20	3
21	3
22	2
23	2
24	3
25	3
26	2
27	2
28	3
29	2
30	2

All components were identified using the EEGLAB extended Infomax Independent Component Analysis (ICA) algorithm (Delorme et al., 2007). Independent Components for eye blinks and lateral eye movements were identified for rejection and subtracted from the data by visual inspection of the component scalp topography, time series, and power spectrum distributions. Note that, unusually, three components were removed from the data of 14 participants. This was done because the ICA algorithm split one of the two typical eye artifact components (one for eye blink and one for lateral eye movements) into two different components. For example, ICA on participant 4's data resulted in a duplication of the typical eye blink component. This high number of participants with three removed components is probably explained by an unusual data lost in frontal electrodes (Fp1, Fpz and/or Fp2) due material deterioration.

Supplementary Table 5 Repeated contrast matrix assigned to the Frequency predictor of each model computed on training blocks.

Condition labels	Intercept	5 *Hz vs.* 1 *Hz* (1st contrast)	10 *Hz vs.* 5 *Hz* (2nd contrast)
1 *Hz*	1	-2/3	-1/3
5 *Hz*	1	1/3	-1/3
10 *Hz*	1	1/3	2/3

Between the three training blocks, we manipulated the Frequency of feedback update at 1, 5 or 10 *Hz*. To relate to our hypotheses testing, we applied the present repeated-contrast matrix to the categorical predictor 'Frequency'. This matrix was obtained by applying the generalised inverse to a *Hypothesis* matrix referring to our hypotheses (Schad et al., 2020). The '5 *Hz vs.* 1 *Hz*' (1st contrast) column relates to the hypothesis that there is a difference in spectral power when participants are presented a feedback updated at 5 *Hz* relative to 1 *Hz*. The '10 *Hz vs.* 5 *Hz*' (2nd contrast) column relates to the hypothesis that there is a difference in spectral power when participants are presented a feedback updated at 10 *Hz* relative to 5 *Hz*.

Supplementary Table 6 Repeated contrast matrix assigned to the Task predictor of each model.

Level labels	Intercept	Alpha-up *vs.* Alpha-Down (1st contrast)	Alpha-Down *vs.* Sham (2nd contrast)
Alpha-Up	1	-2/3	-1/3
Alpha-Down	1	1/3	-1/3
Sham	1	1/3	2/3

Here, we evaluated the effects of both the targeted direction of alpha modulation (i.e., up *vs.* down) and the veracity of the feedback (i.e., genuine *vs.* sham). To this end, we systematically compared the current Alpha-Down group to an Alpha-Up group and to a Sham group, respectively. These two groups were considered from a previous independent double-blind sham-controlled study (Maaz, Waroquier, et al., 2026). To relate to hypothesis testing for both effects, we applied the present matrix to the 'Task' predictor of all models. This matrix was obtained by applying the generalised inverse to the *Hypothesis* matrix referring to the null hypotheses of corresponding effects (Schad et al., 2020). The column of the first contrast ('Alpha-up *vs.* Alpha-Down') relates to the hypothesis of a difference in spectral power when participants are engaged in the up- *vs.* the downregulation of alpha power. The column of the second contrast ('Alpha-Down *vs.* Sham') relates to the hypothesis of a difference in spectral power when submitting participants to a genuine compared to a sham session.

Supplementary Table 7 Estimates from models computed on alpha spectral power during training blocks.

Electrode	Parameter	Estimate	Lower	Upper	BF_{10}	BF_{10+}
**Fz**	**Trial**	**0,018**	**0,012**	**0,024**	**> 100**	**> 100**
Fz	Frequency - 5 Hz *vs*. 1 Hz	0,005	-0,067	0,078	0.037	1.254
Fz	Frequency - 10 Hz *vs*. 5 Hz	0,005	-0,061	0,07	0.033	1.248
Fz	Task - Up *vs*. Down	-0,024	-0,5	0,454	0.24	0.85
Fz	Task - Down *vs*. Sham	0,368	-0,104	0,844	0.796	15.309
Fz	Trial:Frequency - 5 Hz *vs*. 1 Hz	-0,003	-0,015	0,009	0.007	0.453
Fz	Trial:Frequency - 10 Hz *vs*. 5 Hz	0,009	-0,003	0,021	0.017	12.614
Fz	Trial:Task - Up *vs*. Down	-0,002	-0,017	0,012	0.008	0.597
Fz	Trial:Task - Down *vs*. Sham	0,001	-0,013	0,016	0.007	1.32
Fz	Frequency - 5 Hz *vs*. 1 Hz:Task - Up *vs*. Down	-0,12	-0,296	0,056	0.218	0.1
Fz	Frequency - 10 Hz *vs*. 5 Hz:Task - Up *vs*. Down	-0,076	-0,235	0,084	0.124	0.21
Fz	Frequency - 5 Hz *vs*. 1 Hz:Task - Down *vs*. Sham	0,122	-0,055	0,298	0.226	10.526
Fz	Frequency - 10 Hz *vs*. 5 Hz:Task - Down *vs*. Sham	0,01	-0,149	0,171	0.08	1.228
Fz	Trial:Frequency - 5 Hz *vs*. 1 Hz:Task - Up *vs*. Down	0,008	-0,021	0,037	0.017	2.416
Fz	Trial:Frequency - 10 Hz *vs*. 5 Hz:Task - Up *vs*. Down	0,023	-0,006	0,052	0.048	14.872
Fz	Trial:Frequency - 5 Hz *vs*. 1 Hz:Task - Down *vs*. Sham	-0,004	-0,033	0,024	0.016	0.621
Fz	Trial:Frequency - 10 Hz *vs*. 5 Hz:Task - Down *vs*. Sham	-0,025	-0,054	0,005	0.06	0.051
**Cz**	**Trial**	**0,019**	**0,013**	**0,025**	**> 100**	**> 100**
Cz	Frequency - 5 Hz *vs*. 1 Hz	0	-0,072	0,072	0.036	1.022
Cz	Frequency - 10 Hz *vs*. 5 Hz	-0,015	-0,088	0,057	0.04	0.518
Cz	Task - Up *vs*. Down	-0,028	-0,485	0,434	0.236	0.823
Cz	Task - Down *vs*. Sham	0,463	0,003	0,926	1.6	40.082
Cz	Trial:Frequency - 5 Hz *vs*. 1 Hz	-0,002	-0,013	0,009	0.006	0.552
Cz	Trial:Frequency - 10 Hz *vs*. 5 Hz	0,008	-0,005	0,02	0.013	7.607
Cz	Trial:Task - Up *vs*. Down	0,005	-0,01	0,02	0.01	2.837
Cz	Trial:Task - Down *vs*. Sham	-0,001	-0,017	0,014	0.008	0.754
Cz	Frequency - 5 Hz *vs*. 1 Hz:Task - Up *vs*. Down	-0,174	-0,349	0,001	0.603	0.026
Cz	Frequency - 10 Hz *vs*. 5 Hz:Task - Up *vs*. Down	-0,08	-0,256	0,097	0.134	0.224
Cz	Frequency - 5 Hz *vs*. 1 Hz:Task - Down *vs*. Sham	0,122	-0,054	0,296	0.222	10.695
Cz	Frequency - 10 Hz *vs*. 5 Hz:Task - Down *vs*. Sham	0	-0,176	0,177	0.088	1.008
Cz	Trial:Frequency - 5 Hz *vs*. 1 Hz:Task - Up *vs*. Down	0,013	-0,014	0,04	0.021	4.674
Cz	Trial:Frequency - 10 Hz *vs*. 5 Hz:Task - Up *vs*. Down	0,016	-0,015	0,047	0.026	5.63
Cz	Trial:Frequency - 5 Hz *vs*. 1 Hz:Task - Down *vs*. Sham	-0,008	-0,035	0,02	0.016	0.401
Cz	Trial:Frequency - 10 Hz *vs*. 5 Hz:Task - Down *vs*. Sham	-0,022	-0,053	0,009	0.042	0.088
**Pz**	**Trial**	**0,019**	**0,012**	**0,026**	**> 100**	**> 100**
Pz	Frequency - 5 Hz *vs*. 1 Hz	-0,048	-0,137	0,042	0.078	0.174

Pz	Frequency - 10 Hz *vs.* 5 Hz	-0,069	-0,144	0,008	0.19	0.04
Pz	Task - Up *vs.* Down	-0,132	-0,568	0,299	0.262	0.377
**Pz**	**Task - Down *vs.* Sham**	**0,671**	**0,244**	**1,103**	**25.434**	**> 100**
Pz	Trial:Frequency - 5 Hz *vs.* 1 Hz	0,002	-0,012	0,017	0.008	1.688
Pz	Trial:Frequency - 10 Hz *vs.* 5 Hz	0,011	-0,002	0,025	0.027	19.648
Pz	Trial:Task - Up *vs.* Down	0,005	-0,013	0,022	0.01	2.449
Pz	Trial:Task - Down *vs.* Sham	-0,003	-0,021	0,014	0.009	0.554
Pz	Frequency - 5 Hz *vs.* 1 Hz:Task - Up *vs.* Down	-0,26	-0,478	-0,043	1.696	0.01
Pz	Frequency - 10 Hz *vs.* 5 Hz:Task - Up *vs.* Down	-0,018	-0,203	0,167	0.094	0.729
Pz	Frequency - 5 Hz *vs.* 1 Hz:Task - Down *vs.* Sham	0,136	-0,083	0,354	0.232	8.14
Pz	Frequency - 10 Hz *vs.* 5 Hz:Task - Down *vs.* Sham	0,009	-0,177	0,195	0.094	1.16
Pz	Trial:Frequency - 5 Hz *vs.* 1 Hz:Task - Up *vs.* Down	0,034	-0,001	0,069	0.106	32.794
Pz	Trial:Frequency - 10 Hz *vs.* 5 Hz:Task - Up *vs.* Down	0,007	-0,026	0,04	0.018	2.016
Pz	Trial:Frequency - 5 Hz *vs.* 1 Hz:Task - Down *vs.* Sham	-0,021	-0,056	0,015	0.034	0.142
Pz	Trial:Frequency - 10 Hz *vs.* 5 Hz:Task - Down *vs.* Sham	-0,023	-0,056	0,011	0.04	0.101

Each model reported has been computed twice in order to ensure the stability of the BFs. If not specified, each numerical value corresponds to the average of the values obtained across these two model computations. The 'Estimate' column stands for the estimated group-level effects (slopes) of each model 'Parameter' (in z-score standardised units). For the 'Trial' predictor, the estimate corresponds to the group-level effect of one trial of the 1 *Hz* training block (defined as reference for subsequent comparisons for the Frequency predictor) of the Alpha-Up group (defined as reference for subsequent comparisons for the Task predictor). For the 'Frequency' predictor, each comparison (i.e., '5 *Hz vs.* 1 *Hz*' and '10 *Hz vs.* 5 *Hz*') estimate refers to the group-level effect during each training block first trial (modality of Trial predictor defined as reference for subsequent comparisons) of the Alpha-Up group. For the 'Task' predictor, the estimate of both comparisons ('Up *vs.* Down' and 'Down *vs.* Sham') refers to the between-group effect within the first trial of the 1 *Hz* training block. The 'Lower' and 'Upper' columns correspond to the minimal lower and maximal upper bounds of the two 95% CrI computed. The 'BF_{10}' and 'BF_{10+}' columns correspond to the BF in favour of the alternative hypothesis (relative to the null) and the directional (i.e., one-sided) BF, respectively.

Lines in gold highlight corresponding electrodes and parameters for which BFs quantify sufficient evidence in favour of the alternative hypothesis over the null (i.e., presence of an effect) on alpha power.

Supplementary Table 8 Estimates from models computed on theta, SMR and beta spectral power during training blocks.

Frequency Band	Electrode	Parameter	Estimate	Lower	Upper	BF_{10}	BF_{10+}
Theta	Fz	Trial	0,012	0,005	0,019	0.772	> 100
Theta	Fz	Frequency - 5 Hz *vs.* 1 Hz	0,076	0,003	0,15	0.301	47.281
**Theta**	**Fz**	**Frequency - 10 Hz *vs.* 5 Hz**	**-0,108**	**-0,174**	**-0,042**	**4.304**	**0.001**
Theta	Fz	Task - Up *vs.* Down	-0,035	-0,517	0,449	0.246	0.796
Theta	Fz	Task - Down *vs.* Sham	0,208	-0,268	0,682	0.345	4.24
Theta	Fz	Trial:Frequency - 5 Hz *vs.* 1 Hz	-0,014	-0,027	-0,002	0.093	0.01
Theta	Fz	Trial:Frequency - 10 Hz *vs.* 5 Hz	0,016	0,003	0,028	0.136	> 100
Theta	Fz	Trial:Task - Up *vs.* Down	-0,002	-0,02	0,015	0.009	0.679
Theta	Fz	Trial:Task - Down *vs.* Sham	0,016	-0,002	0,033	0.042	23.745

Theta	Fz	Frequency - 5 Hz *vs.* 1 Hz:Task - Up *vs.* Down	0,052	-0,127	0,231	0.106	2.519
Theta	Fz	Frequency - 10 Hz *vs.* 5 Hz:Task - Up *vs.* Down	-0,126	-0,288	0,036	0.268	0.067
Theta	Fz	Frequency - 5 Hz *vs.* 1 Hz:Task - Down *vs.* Sham	0,037	-0,142	0,215	0.098	1.934
Theta	Fz	Frequency - 10 Hz *vs.* 5 Hz:Task - Down *vs.* Sham	0,009	-0,154	0,171	0.082	1.188
Theta	Fz	Trial:Frequency - 5 Hz *vs.* 1 Hz:Task - Up *vs.* Down	-0,014	-0,044	0,016	0.023	0.222
Theta	Fz	Trial:Frequency - 10 Hz *vs.* 5 Hz:Task - Up *vs.* Down	0,024	-0,006	0,054	0.052	15.599
Theta	Fz	Trial:Frequency - 5 Hz *vs.* 1 Hz:Task - Down *vs.* Sham	-0,001	-0,031	0,029	0.016	0.869
Theta	Fz	Trial:Frequency - 10 Hz *vs.* 5 Hz:Task - Down *vs.* Sham	-0,006	-0,036	0,024	0.016	0.549
SMR	Fz	Trial	0,013	0,006	0,02	1.229	> 100
SMR	Fz	Frequency - 5 Hz *vs.* 1 Hz	0,035	-0,041	0,111	0.059	4.55
SMR	Fz	Frequency - 10 Hz *vs.* 5 Hz	0,038	-0,034	0,111	0.064	5.794
SMR	Fz	Task - Up *vs.* Down	-0,114	-0,604	0,369	0.273	0.472
SMR	Fz	Task - Down *vs.* Sham	0,194	-0,289	0,679	0.334	3.656
SMR	Fz	Trial:Frequency - 5 Hz *vs.* 1 Hz	-0,006	-0,019	0,008	0.01	0.266
SMR	Fz	Trial:Frequency - 10 Hz *vs.* 5 Hz	0,005	-0,009	0,019	0.009	3.186
SMR	Fz	Trial:Task - Up *vs.* Down	0,003	-0,015	0,02	0.009	1.659
SMR	Fz	Trial:Task - Down *vs.* Sham	-0,004	-0,021	0,014	0.01	0.495
SMR	Fz	Frequency - 5 Hz *vs.* 1 Hz:Task - Up *vs.* Down	-0,01	-0,195	0,174	0.094	0.841
SMR	Fz	Frequency - 10 Hz *vs.* 5 Hz:Task - Up *vs.* Down	-0,158	-0,333	0,018	0.43	0.039
SMR	Fz	Frequency - 5 Hz *vs.* 1 Hz:Task - Down *vs.* Sham	0,146	-0,038	0,332	0.314	15.794
SMR	Fz	Frequency - 10 Hz *vs.* 5 Hz:Task - Down *vs.* Sham	0,088	-0,087	0,263	0.142	5.136
SMR	Fz	Trial:Frequency - 5 Hz *vs.* 1 Hz:Task - Up *vs.* Down	-0,006	-0,04	0,027	0.018	0.55
SMR	Fz	Trial:Frequency - 10 Hz *vs.* 5 Hz:Task - Up *vs.* Down	0,037	0,003	0,07	0.172	63.902
SMR	Fz	Trial:Frequency - 5 Hz *vs.* 1 Hz:Task - Down *vs.* Sham	-0,002	-0,035	0,032	0.017	0.859
SMR	Fz	Trial:Frequency - 10 Hz *vs.* 5 Hz:Task - Down *vs.* Sham	-0,035	-0,068	-0,001	0.136	0.021
Beta	Fz	Trial	0,008	0	0,016	0.028	37.186
Beta	Fz	Frequency - 5 Hz *vs.* 1 Hz	0,021	-0,078	0,12	0.054	1.969
Beta	Fz	Frequency - 10 Hz *vs.* 5 Hz	-0,082	-0,164	0	0.286	0.026
Beta	Fz	Task - Up *vs.* Down	-0,064	-0,564	0,436	0.259	0.661
Beta	Fz	Task - Down *vs.* Sham	0,084	-0,408	0,583	0.26	1.719
Beta	Fz	Trial:Frequency - 5 Hz *vs.* 1 Hz	0,001	-0,019	0,021	0.01	1.11
Beta	Fz	Trial:Frequency - 10 Hz *vs.* 5 Hz	0,021	0,003	0,039	0.12	81.292
Beta	Fz	Trial:Task - Up *vs.* Down	0,003	-0,018	0,023	0.011	1.498
Beta	Fz	Trial:Task - Down *vs.* Sham	-0,004	-0,024	0,016	0.011	0.557
Beta	Fz	Frequency - 5 Hz *vs.* 1 Hz:Task - Up *vs.* Down	-0,004	-0,243	0,235	0.121	0.954

Beta	Fz	Frequency - 10 Hz *vs.* 5 Hz:Task - Up *vs.* Down	-0,034	-0,233	0,165	0.106	0.581
Beta	Fz	Frequency - 5 Hz *vs.* 1 Hz:Task - Down *vs.* Sham	0,074	-0,165	0,313	0.146	2.717
Beta	Fz	Frequency - 10 Hz *vs.* 5 Hz:Task - Down *vs.* Sham	0,024	-0,175	0,224	0.102	1.46
Beta	Fz	Trial:Frequency - 5 Hz *vs.* 1 Hz:Task - Up *vs.* Down	0,013	-0,036	0,061	0.028	2.292
Beta	Fz	Trial:Frequency - 10 Hz *vs.* 5 Hz:Task - Up *vs.* Down	0,025	-0,02	0,069	0.042	6.298
Beta	Fz	Trial:Frequency - 5 Hz *vs.* 1 Hz:Task - Down *vs.* Sham	-0,021	-0,07	0,028	0.036	0.245
Beta	Fz	Trial:Frequency - 10 Hz *vs.* 5 Hz:Task - Down *vs.* Sham	-0,018	-0,063	0,027	0.031	0.271
Theta	Cz	Trial	0,009	0,002	0,016	0.065	> 100
Theta	Cz	Frequency - 5 Hz *vs.* 1 Hz	0,083	0,012	0,154	0.51	89.811
**Theta**	**Cz**	**Frequency - 10 Hz *vs.* 5 Hz**	**-0,128**	**-0,194**	**-0,062**	**38.64**	**0**
Theta	Cz	Task - Up *vs.* Down	-0,066	-0,542	0,41	0.25	0.646
Theta	Cz	Task - Down *vs.* Sham	0,272	-0,2	0,75	0.454	6.674
Theta	Cz	Trial:Frequency - 5 Hz *vs.* 1 Hz	-0,017	-0,028	-0,007	0.825	0.001
Theta	Cz	Trial:Frequency - 10 Hz *vs.* 5 Hz	0,018	0,007	0,03	0.722	> 100
Theta	Cz	Trial:Task - Up *vs.* Down	-0,005	-0,023	0,012	0.011	0.366
Theta	Cz	Trial:Task - Down *vs.* Sham	0,02	0,003	0,038	0.118	85.283
Theta	Cz	Frequency - 5 Hz *vs.* 1 Hz:Task - Up *vs.* Down	0,014	-0,161	0,188	0.088	1.277
Theta	Cz	Frequency - 10 Hz *vs.* 5 Hz:Task - Up *vs.* Down	-0,17	-0,332	-0,008	0.7	0.02
Theta	Cz	Frequency - 5 Hz *vs.* 1 Hz:Task - Down *vs.* Sham	0,045	-0,129	0,218	0.1	2.277
Theta	Cz	Frequency - 10 Hz *vs.* 5 Hz:Task - Down *vs.* Sham	0,066	-0,097	0,228	0.114	3.742
Theta	Cz	Trial:Frequency - 5 Hz *vs.* 1 Hz:Task - Up *vs.* Down	-0,013	-0,039	0,014	0.021	0.212
Theta	Cz	Trial:Frequency - 10 Hz *vs.* 5 Hz:Task - Up *vs.* Down	0,026	-0,003	0,054	0.071	26.144
Theta	Cz	Trial:Frequency - 5 Hz *vs.* 1 Hz:Task - Down *vs.* Sham	-0,006	-0,033	0,021	0.015	0.487
Theta	Cz	Trial:Frequency - 10 Hz *vs.* 5 Hz:Task - Down *vs.* Sham	-0,009	-0,037	0,019	0.017	0.373
SMR	Cz	Trial	0,01	0,004	0,016	0.616	> 100
SMR	Cz	Frequency - 5 Hz *vs.* 1 Hz	0,022	-0,043	0,086	0.041	2.973
SMR	Cz	Frequency - 10 Hz *vs.* 5 Hz	0,05	-0,014	0,112	0.107	15.653
SMR	Cz	Task - Up *vs.* Down	-0,108	-0,598	0,384	0.27	0.496
SMR	Cz	Task - Down *vs.* Sham	0,236	-0,254	0,732	0.385	4.828
SMR	Cz	Trial:Frequency - 5 Hz *vs.* 1 Hz	-0,004	-0,016	0,008	0.008	0.324
SMR	Cz	Trial:Frequency - 10 Hz *vs.* 5 Hz	0,005	-0,007	0,016	0.008	3.58
SMR	Cz	Trial:Task - Up *vs.* Down	0,005	-0,01	0,02	0.01	3.184
SMR	Cz	Trial:Task - Down *vs.* Sham	-0,006	-0,021	0,009	0.01	0.276
SMR	Cz	Frequency - 5 Hz *vs.* 1 Hz:Task - Up *vs.* Down	-0,088	-0,245	0,069	0.148	0.158
SMR	Cz	Frequency - 10 Hz *vs.* 5 Hz:Task - Up *vs.* Down	-0,165	-0,318	-0,012	0.732	0.017

SMR	Cz	Frequency - 5 Hz *vs.* 1 Hz:Task - Down *vs.* Sham	0,188	0,03	0,345	1.206	99.543
SMR	Cz	Frequency - 10 Hz *vs.* 5 Hz:Task - Down *vs.* Sham	0,096	-0,057	0,249	0.169	8.354
SMR	Cz	Trial:Frequency - 5 Hz *vs.* 1 Hz:Task - Up *vs.* Down	0,001	-0,028	0,03	0.015	1.136
SMR	Cz	Trial:Frequency - 10 Hz *vs.* 5 Hz:Task - Up *vs.* Down	0,03	0,002	0,058	0.132	56.444
SMR	Cz	Trial:Frequency - 5 Hz *vs.* 1 Hz:Task - Down *vs.* Sham	-0,013	-0,042	0,016	0.022	0.237
SMR	Cz	Trial:Frequency - 10 Hz *vs.* 5 Hz:Task - Down *vs.* Sham	-0,026	-0,054	0,003	0.072	0.038
Beta	Cz	Trial	0,002	-0,004	0,008	0.004	2.883
Beta	Cz	Frequency - 5 Hz *vs.* 1 Hz	0,036	-0,04	0,112	0.058	4.655
Beta	Cz	Frequency - 10 Hz *vs.* 5 Hz	-0,045	-0,113	0,023	0.081	0.106
Beta	Cz	Task - Up *vs.* Down	-0,073	-0,579	0,426	0.264	0.637
Beta	Cz	Task - Down *vs.* Sham	0,103	-0,399	0,615	0.274	1.905
Beta	Cz	Trial:Frequency - 5 Hz *vs.* 1 Hz	-0,007	-0,022	0,009	0.011	0.244
Beta	Cz	Trial:Frequency - 10 Hz *vs.* 5 Hz	0,016	0,003	0,03	0.107	> 100
Beta	Cz	Trial:Task - Up *vs.* Down	0,005	-0,01	0,02	0.009	2.681
Beta	Cz	Trial:Task - Down *vs.* Sham	-0,003	-0,018	0,012	0.008	0.512
Beta	Cz	Frequency - 5 Hz *vs.* 1 Hz:Task - Up *vs.* Down	-0,07	-0,253	0,115	0.124	0.294
Beta	Cz	Frequency - 10 Hz *vs.* 5 Hz:Task - Up *vs.* Down	-0,057	-0,223	0,109	0.106	0.33
Beta	Cz	Frequency - 5 Hz *vs.* 1 Hz:Task - Down *vs.* Sham	0,102	-0,083	0,287	0.172	6.302
Beta	Cz	Frequency - 10 Hz *vs.* 5 Hz:Task - Down *vs.* Sham	0,138	-0,028	0,304	0.321	18.933
Beta	Cz	Trial:Frequency - 5 Hz *vs.* 1 Hz:Task - Up *vs.* Down	0,003	-0,035	0,041	0.02	1.32
Beta	Cz	Trial:Frequency - 10 Hz *vs.* 5 Hz:Task - Up *vs.* Down	0,021	-0,012	0,054	0.036	8.392
Beta	Cz	Trial:Frequency - 5 Hz *vs.* 1 Hz:Task - Down *vs.* Sham	-0,02	-0,058	0,018	0.032	0.179
Beta	Cz	Trial:Frequency - 10 Hz *vs.* 5 Hz:Task - Down *vs.* Sham	-0,022	-0,056	0,011	0.04	0.1
Theta	Pz	Trial	0,01	-0,001	0,02	0.027	25.76
Theta	Pz	Frequency - 5 Hz *vs.* 1 Hz	0,06	-0,067	0,186	0.1	4.775
**Theta**	**Pz**	**Frequency - 10 Hz *vs.* 5 Hz**	**-0,179**	**-0,27**	**-0,088**	**70.53**	**0**
Theta	Pz	Task - Up *vs.* Down	-0,223	-0,64	0,194	0.366	0.169
Theta	Pz	Task - Down *vs.* Sham	0,487	0,064	0,908	2.832	82.986
Theta	Pz	Trial:Frequency - 5 Hz *vs.* 1 Hz	-0,013	-0,036	0,01	0.023	0.143
Theta	Pz	Trial:Frequency - 10 Hz *vs.* 5 Hz	0,025	0,006	0,044	0.25	> 100
Theta	Pz	Trial:Task - Up *vs.* Down	-0,018	-0,044	0,009	0.032	0.103
Theta	Pz	Trial:Task - Down *vs.* Sham	0,029	0,003	0,055	0.141	65.488
Theta	Pz	Frequency - 5 Hz *vs.* 1 Hz:Task - Up *vs.* Down	-0,144	-0,45	0,162	0.239	0.214
Theta	Pz	Frequency - 10 Hz *vs.* 5 Hz:Task - Up *vs.* Down	-0,196	-0,419	0,024	0.517	0.042
Theta	Pz	Frequency - 5 Hz *vs.* 1 Hz:Task - Down *vs.* Sham	0,151	-0,155	0,456	0.25	5.087

Theta	Pz	Frequency - 10 Hz *vs.* 5 Hz:Task - Down *vs.* Sham	0,086	-0,135	0,31	0.152	3.565
Theta	Pz	Trial:Frequency - 5 Hz *vs.* 1 Hz:Task - Up *vs.* Down	0,021	-0,034	0,077	0.038	3.482
Theta	Pz	Trial:Frequency - 10 Hz *vs.* 5 Hz:Task - Up *vs.* Down	0,033	-0,014	0,08	0.062	11.124
Theta	Pz	Trial:Frequency - 5 Hz *vs.* 1 Hz:Task - Down *vs.* Sham	-0,042	-0,097	0,014	0.085	0.076
Theta	Pz	Trial:Frequency - 10 Hz *vs.* 5 Hz:Task - Down *vs.* Sham	-0,013	-0,059	0,034	0.027	0.426
SMR	Pz	Trial	0,007	-0,002	0,017	0.015	13.601
SMR	Pz	Frequency - 5 Hz *vs.* 1 Hz	-0,031	-0,143	0,081	0.066	0.413
SMR	Pz	Frequency - 10 Hz *vs.* 5 Hz	0,054	-0,026	0,133	0.096	9.74
SMR	Pz	Task - Up *vs.* Down	-0,142	-0,626	0,338	0.284	0.388
SMR	Pz	Task - Down *vs.* Sham	0,38	-0,103	0,861	0.817	16.093
SMR	Pz	Trial:Frequency - 5 Hz *vs.* 1 Hz	0,002	-0,02	0,025	0.012	1.43
SMR	Pz	Trial:Frequency - 10 Hz *vs.* 5 Hz	0,007	-0,01	0,024	0.012	3.699
SMR	Pz	Trial:Task - Up *vs.* Down	-0,003	-0,026	0,021	0.012	0.69
SMR	Pz	Trial:Task - Down *vs.* Sham	-0,005	-0,028	0,019	0.013	0.52
SMR	Pz	Frequency - 5 Hz *vs.* 1 Hz:Task - Up *vs.* Down	-0,237	-0,508	0,034	0.619	0.044
SMR	Pz	Frequency - 10 Hz *vs.* 5 Hz:Task - Up *vs.* Down	-0,124	-0,318	0,072	0.216	0.117
SMR	Pz	Frequency - 5 Hz *vs.* 1 Hz:Task - Down *vs.* Sham	0,334	0,063	0,602	2.618	> 100
SMR	Pz	Frequency - 10 Hz *vs.* 5 Hz:Task - Down *vs.* Sham	0,11	-0,085	0,305	0.186	6.595
SMR	Pz	Trial:Frequency - 5 Hz *vs.* 1 Hz:Task - Up *vs.* Down	0,025	-0,028	0,079	0.042	4.723
SMR	Pz	Trial:Frequency - 10 Hz *vs.* 5 Hz:Task - Up *vs.* Down	0,023	-0,02	0,065	0.038	5.9
SMR	Pz	Trial:Frequency - 5 Hz *vs.* 1 Hz:Task - Down *vs.* Sham	-0,046	-0,1	0,008	0.113	0.047
SMR	Pz	Trial:Frequency - 10 Hz *vs.* 5 Hz:Task - Down *vs.* Sham	-0,026	-0,069	0,016	0.045	0.124
Beta	Pz	Trial	-0,007	-0,017	0,003	0.014	0.084
Beta	Pz	Frequency - 5 Hz *vs.* 1 Hz	-0,01	-0,134	0,116	0.064	0.786
Beta	Pz	Frequency - 10 Hz *vs.* 5 Hz	-0,033	-0,123	0,057	0.061	0.304
Beta	Pz	Task - Up *vs.* Down	-0,294	-0,777	0,195	0.516	0.13
Beta	Pz	Task - Down *vs.* Sham	0,259	-0,231	0,748	0.437	5.832
Beta	Pz	Trial:Frequency - 5 Hz *vs.* 1 Hz	0,008	-0,017	0,033	0.015	2.72
Beta	Pz	Trial:Frequency - 10 Hz *vs.* 5 Hz	0,015	-0,005	0,035	0.03	13.609
Beta	Pz	Trial:Task - Up *vs.* Down	0,001	-0,022	0,025	0.012	1.195
Beta	Pz	Trial:Task - Down *vs.* Sham	-0,001	-0,025	0,022	0.012	0.855
Beta	Pz	Frequency - 5 Hz *vs.* 1 Hz:Task - Up *vs.* Down	-0,317	-0,618	-0,016	1.286	0.02
Beta	Pz	Frequency - 10 Hz *vs.* 5 Hz:Task - Up *vs.* Down	0,022	-0,197	0,241	0.112	1.383
Beta	Pz	Frequency - 5 Hz *vs.* 1 Hz:Task - Down *vs.* Sham	0,283	-0,018	0,585	0.856	30.16
Beta	Pz	Frequency - 10 Hz *vs.* 5 Hz:Task - Down *vs.* Sham	0,108	-0,11	0,326	0.175	5.017

Beta	Pz	Trial:Frequency - 5 Hz *vs.* 1 Hz:Task - Up *vs.* Down	0,047	-0,014	0,107	0.096	14.338
Beta	Pz	Trial:Frequency - 10 Hz *vs.* 5 Hz:Task - Up *vs.* Down	0,002	-0,046	0,05	0.024	1.122
Beta	Pz	Trial:Frequency - 5 Hz *vs.* 1 Hz:Task - Down *vs.* Sham	-0,058	-0,119	0,002	0.18	0.03
Beta	Pz	Trial:Frequency - 10 Hz *vs.* 5 Hz:Task - Down *vs.* Sham	-0,012	-0,06	0,036	0.028	0.464

Each model reported has been computed twice in order to ensure the stability of the BFs. If not specified, each numerical value corresponds to the average of the values obtained across these two model computations. The 'Estimate' column stands for the estimated group-level effects (slopes) of each model 'Parameter' (in z-score standardised units). For the 'Trial' predictor, the estimate corresponds to the group-level effect of one trial of the 1 *Hz* training block (defined as reference for subsequent comparisons for the Frequency predictor) of the Alpha-Up group (defined as reference for subsequent comparisons for the Task predictor). For the 'Frequency' predictor, each comparison (i.e., '5 *Hz vs.* 1 *Hz*' and '10 *Hz vs.* 5 *Hz*') estimate refers to the group-level effect during each training block first trial (modality of Trial predictor defined as reference for subsequent comparisons) of the Alpha-Up group. For the 'Task' predictor, the estimate of both comparisons ('Alpha-Up *vs.* Alpha-Down' and 'Alpha-Down *vs.* Sham') refers to the between-group effect within the first trial of the 1 *Hz* training block. The 'Lower' and 'Upper' columns correspond to the minimal lower and maximal upper bounds of the two 95% CrI computed. The 'BF_{10}' and 'BF_{10+}' columns correspond to the BF in favour of the alternative hypothesis (relative to the null) and the directional (i.e., one-sided) BF, respectively.
Lines in gold highlight the EEG features for which BFs quantify sufficient evidence in favour of the alternative hypothesis over the null (i.e., presence of an effect).

Supplementary Table 9 Estimates from models computed on theta, SMR and beta spectral power during the transfer block.

**Frequency Band**	**Electrode**	**Parameter**	**Estimate**	**Lower**	**Upper**	***BF*$_{10}$**	***BF*$_{10+}$**
**Theta**	**Fz**	**Trial**	**0,029**	**0,019**	**0,039**	**> 100**	**> 100**
Theta	Fz	Task - Up *vs.* Down	-0,064	-0,567	0,438	0.261	0.666
Theta	Fz	Task - Down *vs.* Sham	0,278	-0,224	0,781	0.47	6.295
Theta	Fz	Trial:Task - Up *vs.* Down	0,003	-0,021	0,028	0.013	1.519
Theta	Fz	Trial:Task - Down *vs.* Sham	0,023	-0,002	0,047	0.066	27.727
**SMR**	**Fz**	**Trial**	**0,022**	**0,012**	**0,033**	**14.988**	**> 100**
SMR	Fz	Task - Up *vs.* Down	-0,07	-0,542	0,401	0.246	0.628
SMR	Fz	Task - Down *vs.* Sham	0,102	-0,371	0,576	0.26	2
SMR	Fz	Trial:Task - Up *vs.* Down	-0,007	-0,033	0,018	0.016	0.4
SMR	Fz	Trial:Task - Down *vs.* Sham	0,039	0,014	0,065	1.122	> 100
Beta	Fz	Trial	0,018	0	0,035	0.064	42.361
Beta	Fz	Task - Up *vs.* Down	-0,012	-0,465	0,441	0.232	0.922
Beta	Fz	Task - Down *vs.* Sham	-0,028	-0,482	0,428	0.231	0.826
Beta	Fz	Trial:Task - Up *vs.* Down	-0,001	-0,043	0,041	0.021	0.927
Beta	Fz	Trial:Task - Down *vs.* Sham	0,023	-0,02	0,065	0.038	6.002
**Theta**	**Cz**	**Trial**	**0,027**	**0,017**	**0,037**	**> 100**	**> 100**
Theta	Cz	Task - Up *vs.* Down	-0,096	-0,603	0,416	0.278	0.548
Theta	Cz	Task - Down *vs.* Sham	0,34	-0,167	0,851	0.607	9.741
Theta	Cz	Trial:Task - Up *vs.* Down	0,005	-0,019	0,029	0.014	2.017
Theta	Cz	Trial:Task - Down *vs.* Sham	0,018	-0,006	0,042	0.036	13.144
**SMR**	**Cz**	**Trial**	**0,016**	**0,008**	**0,024**	**10.94**	**> 100**
SMR	Cz	Task - Up *vs.* Down	-0,032	-0,504	0,45	0.244	0.798
SMR	Cz	Task - Down *vs.* Sham	0,141	-0,336	0,611	0.287	2.622
SMR	Cz	Trial:Task - Up *vs.* Down	-0,007	-0,027	0,013	0.013	0.328
SMR	Cz	Trial:Task - Down *vs.* Sham	0,025	0,006	0,045	0.239	> 100

Beta	Cz	Trial	0,009	-0,001	0,018	0.027	31.083
Beta	Cz	Task - Up *vs.* Down	-0,072	-0,534	0,391	0.242	0.611
Beta	Cz	Task - Down *vs.* Sham	0,035	-0,421	0,489	0.236	1.278
Beta	Cz	Trial:Task - Up *vs.* Down	0,014	-0,009	0,037	0.024	7.913
Beta	Cz	Trial:Task - Down *vs.* Sham	0,015	-0,008	0,038	0.026	8.438
**Theta**	**Pz**	**Trial**	**0,033**	**0,021**	**0,045**	**> 100**	**> 100**
Theta	Pz	Task - Up *vs.* Down	-0,369	-0,857	0,123	0.76	0.075
**Theta**	**Pz**	**Task - Down *vs.* Sham**	**0,653**	**0,16**	**1,144**	**7.065**	**> 100**
Theta	Pz	Trial:Task - Up *vs.* Down	0,005	-0,025	0,034	0.015	1.696
Theta	Pz	Trial:Task - Down *vs.* Sham	0,008	-0,022	0,037	0.017	2.386
SMR	Pz	Trial	0,016	0,007	0,025	1.307	> 100
SMR	Pz	Task - Up *vs.* Down	-0,066	-0,523	0,387	0.238	0.629
SMR	Pz	Task - Down *vs.* Sham	0,25	-0,206	0,702	0.421	6.281
SMR	Pz	Trial:Task - Up *vs.* Down	-0,014	-0,036	0,008	0.024	0.117
SMR	Pz	Trial:Task - Down *vs.* Sham	0,031	0,009	0,053	0.462	> 100
Beta	Pz	Trial	0,006	-0,005	0,016	0.01	6.424
Beta	Pz	Task - Up *vs.* Down	-0,295	-0,729	0,145	0.542	0.1
Beta	Pz	Task - Down *vs.* Sham	0,232	-0,202	0,665	0.388	5.907
Beta	Pz	Trial:Task - Up *vs.* Down	0,005	-0,02	0,03	0.014	1.784
Beta	Pz	Trial:Task - Down *vs.* Sham	0,01	-0,015	0,035	0.017	3.444

Each model reported has been computed twice in order to ensure the stability of the BFs. If not specified, each numerical value corresponds to the average of the values obtained across these two model computations. The 'Estimate' column stands for the estimated group-level effects (slopes) of each model 'Parameter' (in z-score standardised units). For the 'Trial' predictor, the estimate corresponds to the group-level effect of one trial within the Alpha-Up group (defined as reference for subsequent comparisons for the Task predictor). For the 'Task' predictor, the estimate of both comparisons ('Alpha-Up *vs.* Alpha-Down' and 'Alpha-Down *vs.* Sham') refers to the between-group effect within the first trial of the transfer block (defined as reference for subsequent comparisons for the Trial predictor). The 'Lower' and 'Upper' columns correspond to the minimal lower and maximal upper bounds of the two 95% CrI computed. The 'BF_{10}' and 'BF_{10+}' columns correspond to the BF in favour of the alternative hypothesis (relative to the null) and the directional (i.e., one-sided) BF, respectively. Lines in gold highlight the EEG features for which BFs quantify sufficient evidence in favour of the alternative hypothesis over the null (i.e., presence of an effect).

Supplementary Table 10 Completed CRED-nf checklist from Ros et al. (2020).

1. Pre-experiment

a. This experiment was not preregistered.
b. The sample size of the present study was determined using a Bayesian a priori power analysis for three-group comparisons in such experimental design. A sample size of 24 per group was judged sufficient to obtain enough statistical power.

2. Control groups

a. The alpha trajectories of this Alpha-Down group were compared to those of independent Alpha-Up and Sham groups from our previous study (Maaz, Waroquier, et al., 2026), enabling a stringent evaluation of specificity against both genuine but opposite-direction feedback and non-contingent, i.e., sham feedback (Sorger et al., 2019), respectively.
b. The experiment did not directly include a double-blind (only one Alpha-Down group, yet compared to two groups which are included in a double-blind study).
c. Blinding of those who rate the outcome and those who analyse the data:
   - NA: There was only one participant group (data collection).
   - The person who performed all statistical analyses (JM) remained blind to group assignments throughout.

d. Questions aimed to qualitatively assess participants' feeling of feedback control and belief of having received sham feedback, respectively. Importantly, these measures are recommended for implementation in sham-controlled EEG-NF studies to control for differences in feedback and blinding credibility between groups (Ros et al., 2020).
e. NA: This is not a clinical efficacy study

3. Control measures

a. Psychosocial factors were not measured
b. Participants were not informed about the specific EEG-NF target (i.e., downregulate their alpha power at Pz), nor provided with a pre-defined mental strategy. Rather, they were encouraged to find mental strategies correlating with increases in circle size as in common practices (Enriquez-Geppert et al., 2017).
c. The strategies participants used were not recorded or not reported in the manuscript
d. The manuscript does not report the methods used for online-data processing and artifact correction
e. Condition and group effects for artifacts were not measured, or not reported in the manuscript

4. Feedback specifications

a. See the EEG online processing section.
b. See Material and neurofeedback implementation, as well as Procedure sections.
c. During each trial, a grey circle was presented at the centre of a screen. First, during three training blocks, the circle size was continuously updated as real-time feedback of the participant's alpha band (8-12 Hz) power at Pz (Chikhi et al., 2023). The circle size was inversely proportional to real-time alpha power.
d. During three training blocks, the circle size was continuously updated as real-time feedback of the participant's alpha band (8-12 Hz) power at Pz (Chikhi et al., 2023).
e. See Apparatus section.

5. Outcome measures – Brain

a. Concerning the trial repetition effect, extreme evidence was quantified in favour of a positive effect on alpha power.
b. Fig. 1 shows alpha power evolution at frontal, central and parietal electrodes during training depending on the Task participants was submitted to (Alpha-Down EEG-NF, Alpha-Up EEG-NF, or Sham EEG-NF).
   Fig. 3 displays power evolution within each considered band throughout the EEG-NF session.
c. Very strong to extreme evidence was found for the absence of interaction between trial repetition and both the targeted direction of alpha modulation (up vs. down) and feedback veracity (down vs. sham).

6. Outcome measures – Behaviour

a. NA: the study does not take cognitive or behavioural measures.
b. NA: the study does not take cognitive or behavioural measures.

7. Data storage

a. All materials, data and analysis codes are available via the Open Science Framework.

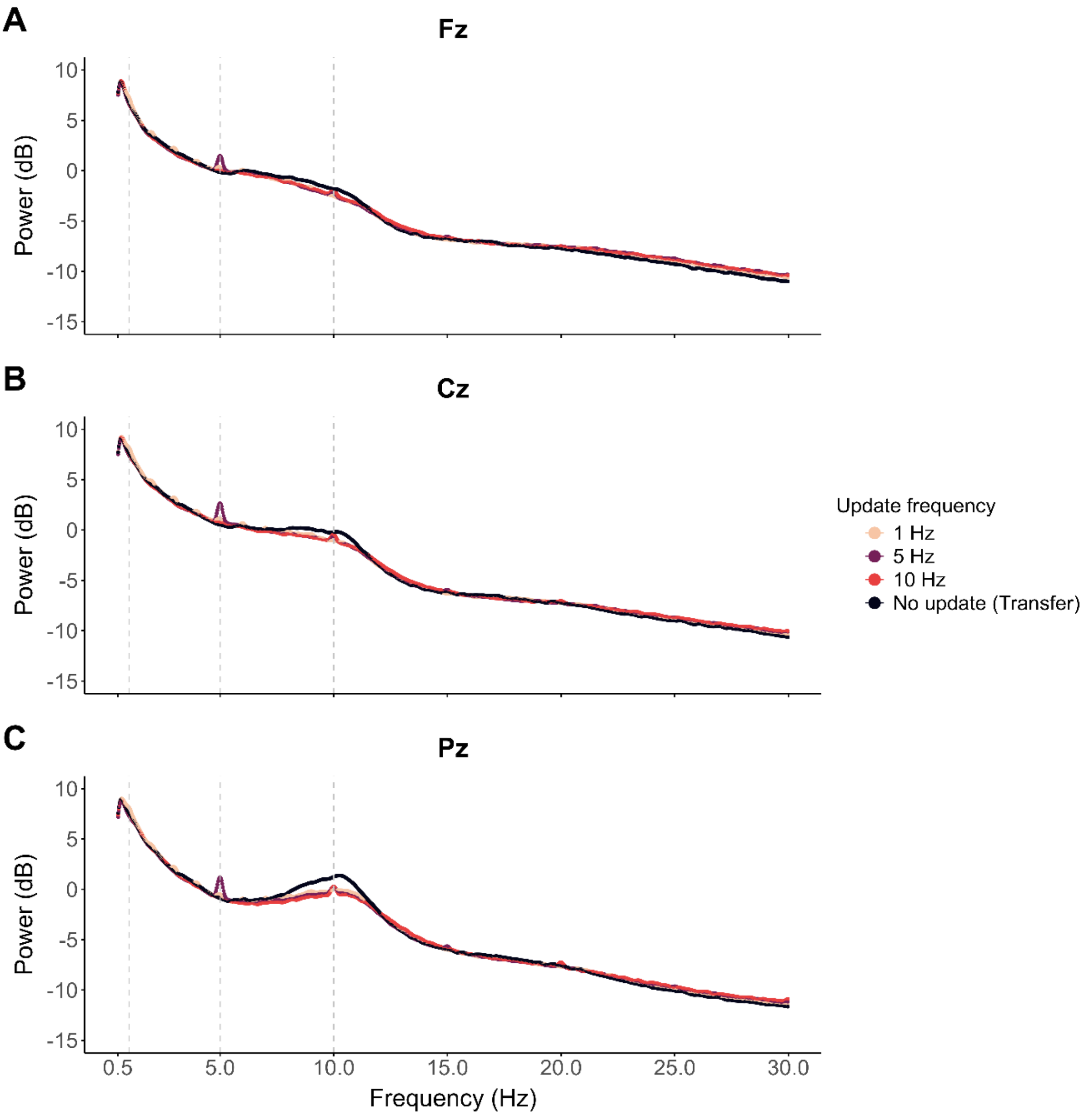

Supplementary Figure 1 Averaged power spectra depending on the frequency of feedback update. Power spectra averaged across groups and trials depending on the feedback update frequency (cream: 1 *Hz*; violet: 5 *Hz*; pink red: 10 *Hz*; black: No update during the Transfer block) and on electrode position (**A**: Fz; **B**: Cz; **C**: Pz). On each panel, vertical dashed lines represent each of the frequencies used for feedback update during training. All spectra have a frequency resolution of ~0.305 *Hz*.

For each electrode, there is a clear peak at 5 *Hz* (i.e., within the theta range of 4-8 *Hz*) when the feedback was updated at this frequency, compared to the other blocks. This suggests that updating the visual feedback at 5 *Hz* entrains an increase in theta spectral power, which was corroborated by statistical analysis (see Supplementary Table 8).

In particular for Pz electrode, one can also notice a smaller peak at 10 *Hz* when using this frequency for feedback update. Even smaller peaks can be identified at 10, 15 and 20 *Hz* when updating feedback at 5 *Hz*, as well as at 20 *Hz* when updating feedback at 10 *Hz*. These may correspond to harmonic responses of the entrainment effects of visual feedback updating at 5 and 10 *Hz*, respectively. These entrainment effects and corresponding harmonics appeared at frequencies comprised within the alpha (8-12 *Hz*) and beta (15-30 *Hz*) bands. One might therefore question whether it would influence their spectral power. However, Bayesian

evidence confirmed the absence of an effect of the frequency of feedback update when comparing these bands absolute spectral power between training blocks (see Supplementary Tables 7-8; although see Supplementary Fig. 2 for a focus on Pz electrode).
Finally, for the averaged spectrum of the Transfer block (black line), no clear peak in power appears, consistent with the absence of rhythmic feedback update during this block. One can also notice stronger averaged power within the alpha band during this block compared to the training blocks (feedback update at 1, 5 or 10 *Hz*). As Transfer systematically followed Training, this corroborates the steady increase in alpha power throughout the present EEG-NF session (see Tables 1-2).

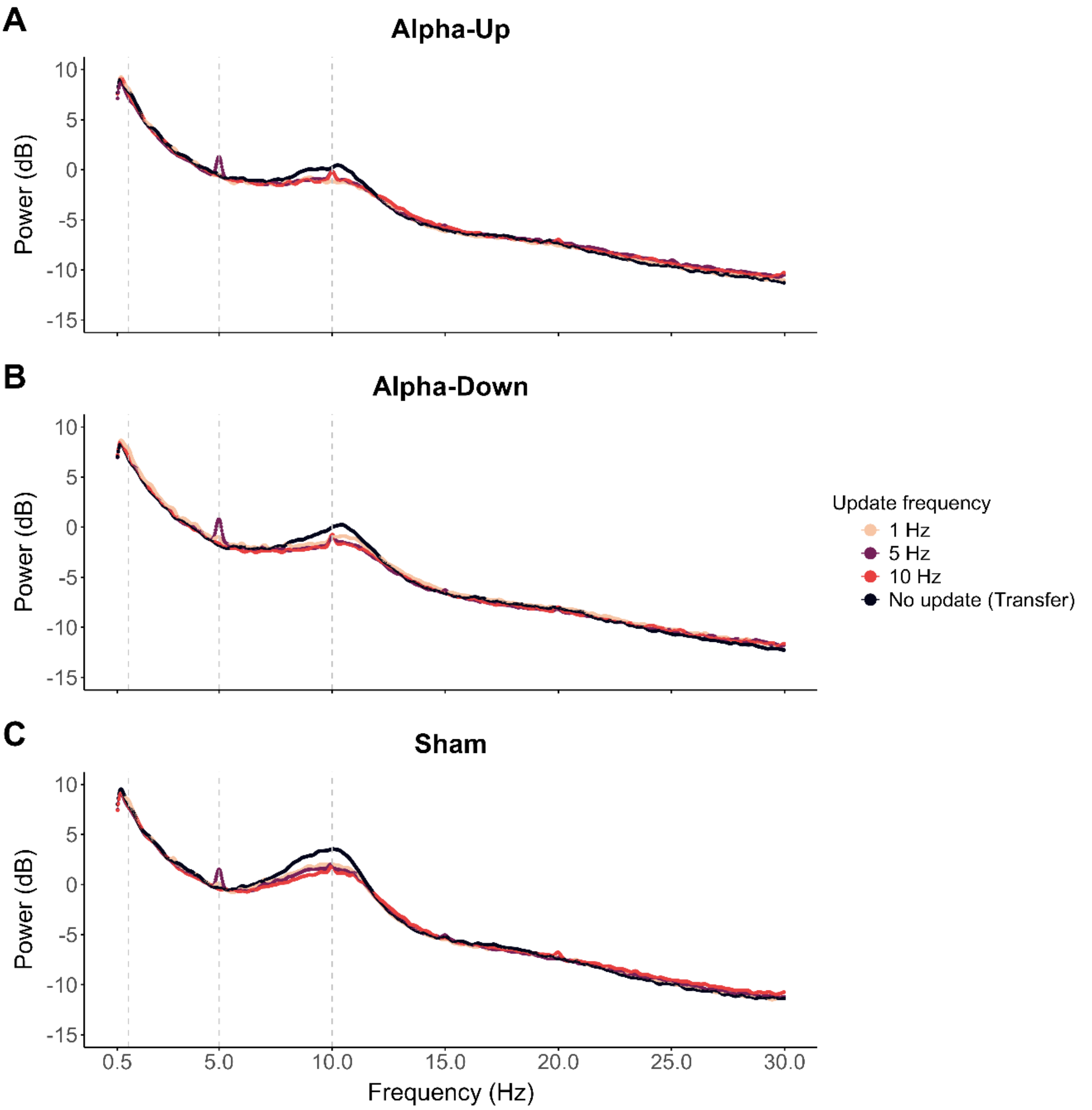

Supplementary Figure 2 Averaged power spectra at Pz depending on participants' Task and on visual feedback display. Power spectra averaged across participants and trials depending on the feedback update frequency (cream: 1 *Hz*; violet: 5 *Hz*; pink red: 10 *Hz*; black: No update during the Transfer block) and on the Task to which participants were submitted to (**A**: Alpha-Up; **B**: Alpha-Down; **C**: Sham). On each panel, vertical dashed lines represent each of the frequencies used for feedback update during training. All spectra have a frequency resolution of ~0.305 *Hz*.

For each Task, there is a clear peak in power at 5 *Hz* when the feedback is updated at this frequency, followed by smaller peaks at 10 and 15 *Hz*. This suggests that updating visual feedback at 5 *Hz* entrains an increase in power at this frequency, as well as possible harmonic responses at 10 and 15 *Hz*. One can thus consider whether this 5 *Hz* entrainment effect artificially increases the spectral power of the theta (4-8 *Hz*) band, and similarly, whether the potential harmonics at 10 and 15 *Hz* entrain an increase of the alpha (8-12 *Hz*) and SMR/beta (12-15/15-30 *Hz*) bands power, respectively. When evaluating absolute difference in power depending on feedback update frequency, very strong evidence supported an increase in theta power when updating feedback at 5 *Hz* compared to 10 *Hz*. However, Bayesian analyses

confirmed the absence of an effect of the feedback update frequency on absolute alpha, SMR and beta power (see Supplementary Tables 7-8).

Similarly, updating the feedback at 10 *Hz* seems to entrain an increase in power at this frequency (and possible harmonic at 20 *Hz*) in all groups. However, statistical analyses confirmed the absence of an effect on the absolute power of the alpha and beta frequency bands (see Supplementary Tables 7-8).
